# DrugRepoBank: a comprehensive database and discovery platform for accelerating drug repositioning

**DOI:** 10.1093/database/baae051

**Published:** 2024-07-11

**Authors:** Yixian Huang, Danhong Dong, Wenyang Zhang, Ruiting Wang, Yang-Chi-Dung Lin, Huali Zuo, Hsi-Yuan Huang, Hsien-Da Huang

**Affiliations:** School of Medicine, The Chinese University of Hong Kong, Shenzhen, Longgang District, Shenzhen, Guangdong 518172, China; Warshel Institute for Computational Biology, School of Medicine, The Chinese University of Hong Kong, Shenzhen, Longgang District, Shenzhen, Guangdong 518172, China; School of Medicine, The Chinese University of Hong Kong, Shenzhen, Longgang District, Shenzhen, Guangdong 518172, China; Warshel Institute for Computational Biology, School of Medicine, The Chinese University of Hong Kong, Shenzhen, Longgang District, Shenzhen, Guangdong 518172, China; School of Medicine, The Chinese University of Hong Kong, Shenzhen, Longgang District, Shenzhen, Guangdong 518172, China; Warshel Institute for Computational Biology, School of Medicine, The Chinese University of Hong Kong, Shenzhen, Longgang District, Shenzhen, Guangdong 518172, China; School of Medicine, The Chinese University of Hong Kong, Shenzhen, Longgang District, Shenzhen, Guangdong 518172, China; Warshel Institute for Computational Biology, School of Medicine, The Chinese University of Hong Kong, Shenzhen, Longgang District, Shenzhen, Guangdong 518172, China; School of Medicine, The Chinese University of Hong Kong, Shenzhen, Longgang District, Shenzhen, Guangdong 518172, China; Warshel Institute for Computational Biology, School of Medicine, The Chinese University of Hong Kong, Shenzhen, Longgang District, Shenzhen, Guangdong 518172, China; School of Medicine, The Chinese University of Hong Kong, Shenzhen, Longgang District, Shenzhen, Guangdong 518172, China; Warshel Institute for Computational Biology, School of Medicine, The Chinese University of Hong Kong, Shenzhen, Longgang District, Shenzhen, Guangdong 518172, China; School of Medicine, The Chinese University of Hong Kong, Shenzhen, Longgang District, Shenzhen, Guangdong 518172, China; Warshel Institute for Computational Biology, School of Medicine, The Chinese University of Hong Kong, Shenzhen, Longgang District, Shenzhen, Guangdong 518172, China; School of Medicine, The Chinese University of Hong Kong, Shenzhen, Longgang District, Shenzhen, Guangdong 518172, China; Warshel Institute for Computational Biology, School of Medicine, The Chinese University of Hong Kong, Shenzhen, Longgang District, Shenzhen, Guangdong 518172, China

## Abstract

In recent years, drug repositioning has emerged as a promising alternative to the time-consuming, expensive and risky process of developing new drugs for diseases. However, the current database for drug repositioning faces several issues, including insufficient data volume, restricted data types, algorithm inaccuracies resulting from the neglect of multidimensional or heterogeneous data, a lack of systematic organization of literature data associated with drug repositioning, limited analytical capabilities and user-unfriendly webpage interfaces. Hence, we have established the first all-encompassing database called DrugRepoBank, consisting of two main modules: the ‘Literature’ module and the ‘Prediction’ module. The ‘Literature’ module serves as the largest repository of literature-supported drug repositioning data with experimental evidence, encompassing 169 repositioned drugs from 134 articles from 1 January 2000 to 1 July 2023. The ‘Prediction’ module employs 18 efficient algorithms, including similarity-based, artificial-intelligence-based, signature-based and network-based methods to predict repositioned drug candidates. The DrugRepoBank features an interactive and user-friendly web interface and offers comprehensive functionalities such as bioinformatics analysis of disease signatures. When users provide information about a drug, target or disease of interest, DrugRepoBank offers new indications and targets for the drug, proposes new drugs that bind to the target or suggests potential drugs for the queried disease. Additionally, it provides basic information about drugs, targets or diseases, along with supporting literature. We utilize three case studies to demonstrate the feasibility and effectiveness of predictively repositioned drugs within DrugRepoBank. The establishment of the DrugRepoBank database will significantly accelerate the pace of drug repositioning.

**Database URL:** https://awi.cuhk.edu.cn/DrugRepoBank

## Introduction

Drug repositioning, also referred to as drug repurposing or drug re-tasking, is a strategic approach that aims at identifying new therapeutic applications for existing drugs. This innovative approach uncovers the new indication potential of drugs that have already been approved, discontinued, abandoned or in experimental stages (1, 2). Compared with the time-consuming, expensive and high-risk process of traditional drug discovery, drug repositioning offers a novel pathway to address these challenges, thereby revolutionizing the drug development landscape. It directly addresses the high failure rate of approximately 45% attributed to safety and toxicity concerns in traditional drug discovery programs. Additionally, it has the potential to reduce the drug development timeline by an average of 5–7 years (3, 4). It has been accounted that approximately 30% of the US Food and Drug Administration (FDA)-approved drugs are repositioned drugs. Some of the most successful and best-known drugs that come from the drug repositioning approach are sildenafil, minoxidil, aspirin, valproic acid, methotrexate, etc. (5). For instance, sildenafil, initially developed for hypertension and angina pectoris, was successfully repositioned to treat erectile dysfunction. Drug repositioning plays a pivotal role in pharmaceutical innovation due to its ability to expedite drug development, minimize costs and decrease the likelihood of failure. It bypasses the initial stages of drug discovery, leveraging existing data on pharmacokinetics, toxicology and clinical safety, and can redirect efforts toward tackling infectious diseases, hard-to-treat illnesses and neglected diseases. This strategic shift not only revitalizes existing compounds but also accelerates the path to finding effective treatments for a broader array of health conditions.

Recently, several databases have appeared, aiming to explore the potential of repositioned drugs (Table 1). Connectivity Map (CMap) (6), published in 2006, contains over 1 million gene expression signatures associated with various perturbations. These signatures can be employed to elucidate relationships between drugs, genes and diseases. By comparing differential expression signatures induced by diseases with drug treatment signatures in the CMap, connectivity scores can be derived to rank these relationships. These connections can serve as a foundation for formulating hypotheses related to disease treatments. However, CMap has limitations, including a shortage of drug perturbation data, limited drug coverage, dosage-dependent conditions and the uncertainty associated with applying cell line or animal model expression patterns to human body systems. Promiscuous (7), established in 2010, encompasses an extensive dataset comprising approximately 25 000 drugs, 6500 targets, around 21 500 drug-target interactions and information on approximately 1100 side effects. This database employs similarity-based and network-based methodologies for drug repositioning and has demonstrated its utility in identifying potential candidates. However, Promiscuous contains a relatively limited amount of data, and the accuracy of its predictive algorithms requires further computational and experimental validation. The DrugSig resource (8), introduced in 2017, involves the comparison of disease gene expression signatures with the signatures of known drugs for drug repositioning. Currently, DrugSig includes only 1300 drugs and over 6000 signatures. However, the database’s functionalities and methods are somewhat limited. There is a need to integrate additional gene function analysis tools and other computational drug repurposing approaches into DrugSig to enhance its user interactivity. RepoDB (9), launched in 2017, serves as a database that catalogs approved and failed drugs and their respective indications. However, it lacks additional information or features. This limitation hinders users from performing further analyses. RepurposeDB (10), unveiled in 2018, offers a comprehensive collection and analysis of drug repositioning cases, assembles successfully repositioned drugs and presents a set of principles grounded in pharmacology, biology and disease specificity for drug repositioning. Nonetheless, RepurposeDB lacks target prediction capabilities and an interactive visualization module. It also has a relatively limited dataset, comprising approximately 250 drugs and 300 targets. Experimental Knowledge-Based Drug Repositioning Database (EK-DRD) (11), launched in 2019, is a database that aggregates experimental knowledge-based information for drug repositioning. However, it relies solely on information related to the drug, such as known targets or scientific articles describing areas of repositioning research for that drug. Furthermore, it does not conduct prediction analysis such as similarity searches or similar analyses, rendering its functionality inapplicable to newly derived structures. Promiscuous 2.0 (12), introduced in 2020, represents an enhanced iteration of Promiscuous, featuring a substantial increase in data. It includes an expanded compound count to 1 million and a significant rise in drug-target interactions to 2 727 520. It also introduces disease data and incorporates the capability to predict targets through similarity-based and machine-learning-based methods, addressing some of the limitations of the original Promiscuous. However, it is important to note that similarity and machine learning methods rely on compound structural features while overlooking other factors, such as target protein and related pathways of the query drugs, which may impact prediction accuracy. LINCS (Library of Integrated Network-Based Cellular Signatures) Data Portal 2.0 (13), released in 2020, provides a comprehensive catalog of 21 231 perturbation-response signatures by utilizing a diverse collection of perturbations across many model systems and assay types. While LINCS offers a greater amount of signature data than CMap, there is still room for improvement in the accuracy and speed of its algorithms. NeDRex (14), launched in 2021, serves as an integrative and interactive platform designed for network-based drug repurposing and the discovery of disease modules. It consolidates data from ten sources encompassing genes, drugs, drug targets, disease annotations and their interrelationships. NeDRex enables the construction of heterogeneous biological networks, exploration for disease modules and prioritization of drugs targeting disease mechanisms, and includes statistical validation processes. However, it is important to acknowledge that the network-based algorithm used by NeDRex has specific limitations, such as false-positive protein–protein interactions (PPIs), potential literature bias stemming from under- and over-studied genes and the limitation that drug–protein associations within the integrated databases may not distinguish between activation and inhibition. DrugSimDB (15), introduced in 2021, provides comprehensive information about drugs and targets while integrating multiple similarity-based approaches for drug repositioning to enhance the credibility of its similarity method. Nevertheless, there is room for further optimization of the similarity algorithm used by DrugSimDB, such as incorporating a broader range of drug-related information to accommodate a wider array of drug categories or proteins. DREIMT (16), introduced in 2021, integrates 4690 drug consensus profiles and over 2600 immune gene expression signatures to establish associations between drugs and immune signatures. However, DREIMT is currently constrained in its scope, primarily focused on repurposing existing drugs for immunomodulatory diseases, which restricts its application to other medical conditions. PharmOmics (17), launched in 2022, is a species- and tissue-specific database encompassing 13 530 transcriptomic datasets from rats, humans and mice across more than 20 tissues, spanning 941 drugs. Currently, the network-based algorithms employed by PharmOmics are limited by the scope of predefined tissue-specific regulatory networks.

**Table 1.** Comparison of DrugRepoBank with other 12 drug repositioning databases

	Data amount and features	DrugRepoBank	PharmOmics	DREIMT	DrugSimDB	NeDRex	LINCS Data Portal 2.0	Promiscuous 2.0	EK-DRD	RepurposeDB	repoDB	DrugSig	Promiscuous	Connectivity map
Database	Drugs	49 652	941	0	10 317	13 300	21 231	991 805	1963	253	1571	1300+	25 000	5000
	Targets	4221	18 710	70	0	212 745	0	9430	2985	305	0	800	6500	0
	Drug–target interactions	880 945	0	0	20 061	29 932	0	2 727 520	30 944	0	0	0	21 500	0
	Drug–disease interactions	28 978	0	0	0	0	0	0	0	0	6677	0	0	0
	Drug signature	473 647	14 366	4694	0	36 025	570 862	0	0	0	0	6000	0	1 500 000
	Disease signature	25	0	2687	0	24 120	0	0	0	0	0	0	0	0
	Pathway	6700	0	0	3888	2309	0	0	1332	0	0	0	1600	0
	Side effect	109 698	0	0	139 756	0	0	4964	0	0	0	0	1100	0
	Disease	3379	0	0	10 562	0	0	3379	856	1125	2051	0	0	0
	Predicted targets	Yes	0	0	0	0	0	Yes	0	0	0	0	0	0
	Literature	Yes	No	No	No	No	No	No	Yes	No	No	No	No	No
Network	Network visualization	Interactive	Interactive	None	Interactive	Interactive	None	Interactive	Static	Static	None	None	Interactive	None
Annotation	Functional enrichment analysis	Yes	Yes	Yes	No	Yes	Yes	No	No	Yes	No	No	No	No
Prediction algorithms	Similarity- based algorithm	Yes	No	No	Yes	No	No	Yes	Yes	No	No	No	Yes	No
	Artificial-intelligence- based algorithm	Yes	No	No	No	No	No	Yes	No	No	No	No	No	No
	Signature- based algorithm	Yes	Yes	Yes	No	No	Yes	No	No	No	No	Yes	No	Yes
	Network- based algorithm	Yes	Yes	No	No	Yes	No	No	No	No	No	No	Yes	No
Publications	Year		2022	2021	2021	2021	2020	2020	2019	2018	2017	2017	2010	2006
	Publish journal		iScience	Bioinformatics	Briefing in Bioinformatics	Nature Commun-ications	Nucleic Acids Research	Nucleic Acids Research	Journal of Chemical Information and Modeling	Briefings in Bioinformatics	Scientific data	PLoS One	Nucleic acids Research	Science
	Number of citations (Accessed Google Scholar on 1 September 2023)		5	9	9	28	103	17	5	78	171	47	244	4848
Web interface	Link	https://awi.cuhk.edu.cn/DrugRepoBank	http://mergeomics.research.idre.ucla.edu	http://www.dreimt.org/	http://vafaeelab.com/drugSimDB.html	https://api.nedrex.net	https://lincsportal.ccs.miami.edu/signatures/home	http://bioinformatics.charite.de/promiscuous2	https://www.idruglab.com/drd/index.php	http://repurposedb.dudleylab.org/	http://apps.chiragjpgroup.org/repoDB/	http://biotechlab.fudan.edu.cn/database/drugsig/	http://bioinformatics.charite.de/promiscuous	https://portals.broadinstitute.org/cmap
	Accessibility	Yes	Yes	Yes	Yes	Yes	Yes	Yes	No	Yes	Yes	No	Yes	Yes
Reference			(17)	(16)	(15)	(14)	(13)	(12)	(11)	(10)	(9)	(8)	(7)	(6)

In summary, despite the existence of numerous databases dedicated to drug repositioning, several issues persist within them, including inadequate data volume, limited data types, inaccuracies in algorithms due to the lack of consideration for multidimensional or heterogeneous data, the absence of systematic collation of literature data related to drug repositioning, limited analytical capabilities and user-unfriendly webpage interfaces. These recurring issues highlight the urgent need for an integrated platform in this area.

**Figure 1.**
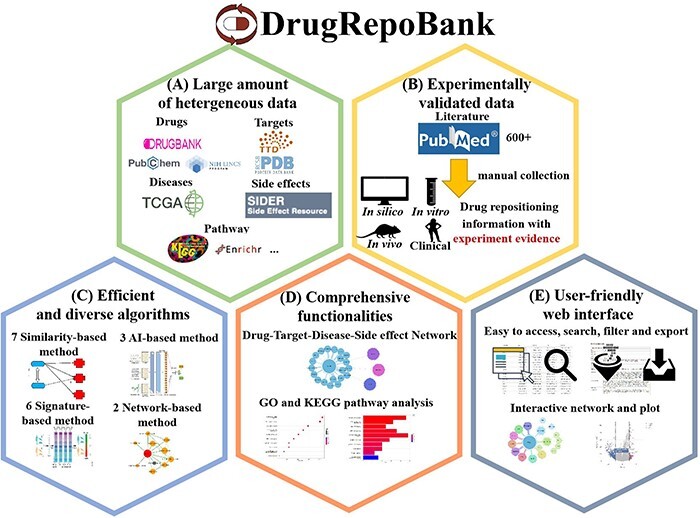
Characteristics of DrugRepoBank. (A) A large amount of heterogeneous data (drugs, targets, diseases, side effects and pathways) was integrated from several high-quality databases. (B) Experimentally validated drug repositioning information manually collected from the literature. (C) Multi-dimension algorithms include seven similarity-based methods, six signature-based methods, two network-based method and three artificial-intelligence-based methods. (D) Comprehensive functionalities to explore biological significance include network visualization of drug-target-disease-side effect network, pathway analysis of disease signatures, etc. (E) A user-friendly web interface makes the database easy to access, search, filter and export.

In response, we propose DrugRepoBank, a novel and comprehensive repository and discovery platform for drug repositioning (depicted in Figure 1). This study showcases DrugRepoBank’s distinguishing features and its clear superiority over preceding databases:

It aggregates an extensive volume of heterogeneous data encompassing drugs, targets, diseases, side-effects and pathways, sourced from multiple high-quality databases.

The platform includes experimentally validated drug repositioning information, meticulously curated from the literature through manual extraction.

Leveraging state-of-the-art methodologies, it implements multi-dimensional algorithms such as seven similarity-based methods, three artificial intelligence-based methods, six signature-based methods and two network-based methods, thereby addressing the inadequacies in existing computational models.

With a suite of comprehensive functionalities, DrugRepoBank enables users to delve into the biological implications with tools like network visualization of drug-target-disease-side effect networks, pathway analyses for disease signatures and so on.

(E) The database boasts a highly user-friendly web interface that facilitates seamless access, searching, filtering and exporting capabilities, significantly enhancing user experience.

Considering the escalating prominence of drug repositioning in modern drug discovery strategies, our freshly established DrugRepoBank database (available at https://awi.cuhk.edu.cn/DrugRepoBank) is expected to wield considerable influence on the trajectory and efficiency of future drug exploration efforts.

## Materials and methods

### Data collection and processing

#### Manual curation of literature-supported repositioned drugs with experimental evidence

**Figure 2.**
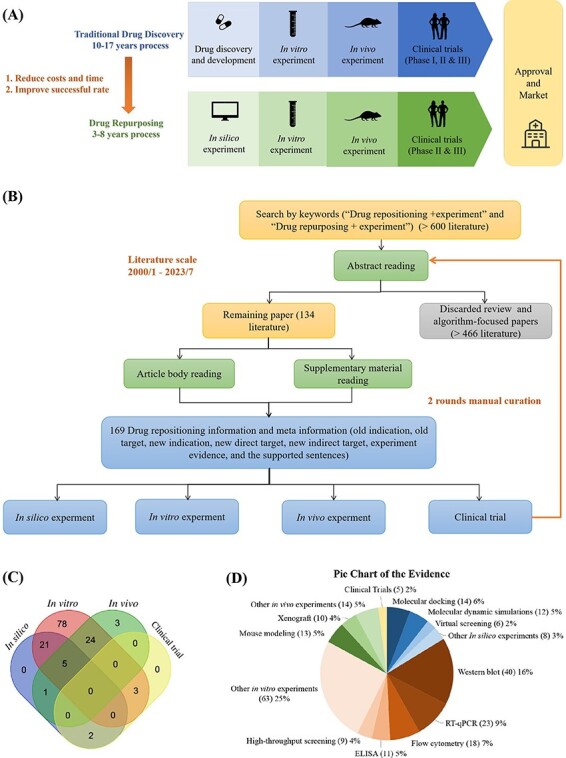
Manually curated experimentally validated repositioned drugs from published literature. (A) Drug repositioning stages compared with drug discovery process. (B) Literature search and data collection procedures. (C)Venn diagram of the number of repositioning drugs in four experimental stages. (D) The Pie Chart is drawn from the experiment evidence of 134 literature which contains drug repositioning information. Experimental evidence encompasses four primary categories: “*in silico* experiments”, “*in vitro* experiments”, “*in vivo* experiments”, and “clinical trials”.

Drug repositioning represents a highly efficient strategy for identifying established pharmaceutical agents with the potential to treat emerging diseases effectively. As depicted in Figure 2A, the drug repositioning methodology significantly reduces time requirements compared to the conventional drug discovery pathway. Specifically, the drug repositioning process is categorized into four pivotal stages: *in silico* experimentation, *in vitro* experimentation, *in vivo* validation and clinical trial assessment. These stages collectively comprise a progressively ascending hierarchy of evidentiary substantiation.

To systematically collect and analyze data on drug repositioning in the literature, we developed the ‘Literature’ module in DrugRepoBank, an experimentally validated drug repositioning database through a manual curation approach from the PubMed database. Optimized keyword combinations of ‘drug repositioning + experiment’ and ‘drug repurposing + experiment’ were applied in the PubMed Advanced Search Builder tool, and we kept searched results in the limited time range between January 2000 and July 2023, resulting in >600 research articles. To ensure the reliability of the data, we conducted two rounds of literature checks (Figure 2B). We initially reviewed the abstracts of these papers to exclude review articles and algorithm-focused papers and eventually obtained 134 valid literature sources. We further read the article body and supplementary materials to obtain 169 experimentally validated repositioned drugs and corresponding meta-information, including old indication (the original medical condition or disease for which a drug was initially developed and approved), old target (a protein that the drug was originally designed to interact with or modulate for its initial therapeutic purpose), new indication (a different medical condition or therapeutic use for a drug that was originally developed and approved for a specific purpose), new direct target (the novel or alternative molecular target that the drug affects in the context of treating a different disease or medical condition), new indirect target (a component of a pathway or mechanism that may not be the primary intended target of the drug but is relevant to its efficacy in treating a different disease or medical condition), experiment evidence (such as qPCR, Western Blot, etc.) and the supporting sentences in literature. To effectively demonstrate the drug repositioning evidence levels, we have categorized the collected evidence for each drug into four distinct levels: *in silico* experiments (computation level), *in vitro* experiments (cell level), *in vivo* experiments (animal level) and clinical trials (human body level). Each of these levels provides a varying degree of support for drug repositioning hypotheses.

#### Integrated databases

To provide users with comprehensive information on drug repositioning, DrugRepoBank integrates multiple types of drug repositioning-related data shown in Table 2.

**Table 2.** Data class, specific data, statistics and details of data sources used to generate DrugRepoBank

Data class	Specific data	Statistics	Details	Data source	Ref
Drug	Drug name, clinical status and drug identifiers	49 652 small-molecule drugs including 2877 approved drugs	DrugBank ID, drug name, Drug clinical status, drug identifiers including CAS Number, InChIKey, InChI, Formula and SMILES	DrugBank	(18)
	Drug chemical structures	29 733 structures	We use the PubChem Compound ID of drugs to search the drug chemical structures in MolView	PubChem	(19)
Drug–target interaction	Drug protein targets	880 945 drug–target interactions	Each drug–target pair records the mechanism of action (such as agonist, antagonist and so on) and activity (Kd, Ki or IC50).	DrugBank and TTD	(18, 20, 21)
Drug-disease association	Drug indications and clinical status	28 978 drug-indication associations, including 3620 ‘approved’	Drug disease indications are encoded by ICD-11. The clinical status includes Phase 1, Phase 1/2, Phase 2, Phase 3, Approved, Terminated, Investigated, Discontinued in Phase 1, Discontinued in Phase 2, Discontinued in Phase 3, Patented, Withdraw from market, Preclinical, Discontinued in Preregistration and Clinical trial	TTD	(20, 21)
Drug-side effects association	Drug-side effects association	109 698 Drug-side effect associations	Information on marketed medicines and their recorded adverse drug reactions	SIDER	(22)
Drug-pathway association	Drug-induced pathways	243 pathways and 3888 drug-pathway associations		KEGG	(23)
Target	Target name, sequence, target identifiers and other target information	4221 targets, including 620 successful targets	Target name, target gene name, target type, synonyms, biochemical class, EC Number and sequence	TTD	(20, 21)
	Target 3D structure	7082 structures of 2226 targets	We use PDB ID to search target 3D structures in https://molstar.org.	PDB	(29)
Target-disease association	Target indications and clinical status	11 268 target-indication associations, including 2679 ‘approved’	Target disease indications are encoded by ICD-11. The clinical status includes Phase 1, Phase 1/2, Phase 2, Phase 3, Approved, Terminated, Investigated, Discontinued in Phase 1, Discontinued in Phase 2, Discontinued in Phase 3, Patented, Withdraw from market, Preclinical, Discontinued in Preregistration and Clinical trial	TTD	(20, 21)
Target-pathway association	Target-involved KEGG pathways	387 pathways and 8528 target-pathway associations	We provide target-involved KEGG pathways for Homo sapiens	KEGG	(23)
	Target-involved WiKi pathways	516 pathways and 8149 target-pathway associations		WiKiPathways	(30)
	Target-involved PathWhiz pathways	98 pathways and 467 target-pathway associations		PathWhizPathway	(31)
	Target-involved Reactome pathways	577 pathways and 4332 target-pathway associations		REACTOME	(32)
	Target-involved NetPath pathways	25 pathways and 1106 target-pathway associations		NetPath	(33)
	Target-involved PANTHER pathways	124 pathways and 1786 target-pathway associations		PANTHER	(34)
Pathway	GO terms and annotations	6700 GO terms, including 446 CC, 1151 MF and 5103 BP terms, and a total of 250 734 protein-GO term associations	GO terms across categories of cellular components (CC), molecular functions (MF) and biological process (BP)	Enrichr	(35)
Disease signature	Diseases (Cancer) signature	25 types of cancer	Breast Cancer (BRCA), Bladder Cancer (BLCA), Cervical Cancer (CESC), Bile Duct Cancer (CHOL), Colon Cancer (COAD), Colon and Rectal Cancer (COADREAD), Esophageal Cancer (ESCA), Head and Neck Cancer (HNSC), Kidney Chromophobe (KICH), Kidney Clear Cell Carcinoma (KIRC), Kidney Papillary Cell Carcinoma (KIRP), Liver Cancer (LIHC), Lung Adenocarcinoma (LUAD), Lung Cancer (LUNG), Lung Squamous Cell Carcinoma (LUSC), Pancreatic Cancer (PADD), Pheochromocytoma & Paraganglioma (PCPG), Prostate Cancer (PRAD), Rectal Cancer (READ), Sarcoma (SARC), Melanoma (SKCM), Stomach Cancer (STAD), Thyroid Cancer (THCA), Thymoma (THYM) and Endometrioid Cancer (UCEC)	TCGA	(36)
Drug signature	Drug signature	473 647 replicate-consensus signatures	We downloaded the level 5 data of L1000 (GCTx format) from the Gene Expression Omnibus (accession number: GSE92742), which contains 473 647 replicate-consensus signatures (RCSs) generated by the official data pre-processing pipeline. The level 5 data of L1000 have been normalized, and the LINCS team suggests their direct use without extra processing. Each RCS represents the moderated z-score value of 12 328 genes for one profile	L1000	(37)
Literature	Drug repositioning-related literature	169 experimentally validated repositioned drugs from 134 valid literature	We extract important information such as old targets, new direct targets, new indirect targets, old diseases, new diseases, experiment evidence and supporting sentences from these articles	PubMed	(38)

As for drug-centered information, the database encompasses 49 652 small-molecule drugs, including 2877 approved ones, with detailed information such as names, clinical statuses and various identifiers (e.g. DrugBank ID, CAS Number, InChIKey) sourced from DrugBank (18). The platform also provides access to 29 733 chemical structures of these drugs, retrieved from the PubChem database (19) using PubChem Compound IDs via MolView. Besides, DrugRepoBank documents 880 945 drug-target interactions, specifying the mechanisms of action (agonist, antagonist, etc.) and activity values (Kd, Ki, IC50) for each drug–protein pair, drawing from both DrugBank (18) and Therapeutic Target Database (TTD) (20, 21). Furthermore, it also catalogs 28 978 drug-indication associations, with 3620 marked as ‘approved’, encoding disease indications using ICD-11 and listing exhaustive clinical phases and statuses (Supplementary Table S1) from TTD (20, 21) and the official site of ClinicalTrial.gov. Additionally, it includes 109 698 drug-side effect associations sourced from SIDER (22) to highlight potential safety concerns. In terms of drug-pathway associations, DrugRepoBank features 243 pathways influenced by drugs and 3888 corresponding drug-pathway associations from KEGG (23). In order to offer a holistic understanding for drugs through various aspects, we have also provided hyperlinks that directly connect users to a range of additional drug-related databases such as DrugMAP (24), DRESIS (25), TheMarker (26), VARIDT (27) and INTEDE (28) for exploring molecular interactions, resistance mechanisms, biomarkers, drug transporters and metabolic pathways, respectively.

For target-centered information, the database covers 4221 targets, including 620 successful ones, providing target names, sequences, gene names, types, synonyms, biochemical classes, EC Numbers and other relevant details from TTD (20, 21). Users can access the 3D structures of 2226 targets through 7082 structures sourced from the Protein Data Bank (PDB) (29) available on https://molstar.org using PDB IDs. Moreover, it presents 11 268 target-indication associations, of which 2679 are ‘approved’, again utilizing ICD-11 disease coding and detailing clinical stages from TTD (20, 21). The platform further explores target-pathway relationships by incorporating target involvements in various databases: KEGG (23), WikiPathways (30), PathWhiz (31), Reactome (32), NetPath (33) and PANTHER (34), offering a comprehensive view of target participation in biological pathways across multiple resources. DrugRepoBank also includes 6700 GO terms spanning cellular components (CC), molecular functions (MF) and biological processes (BP), along with 250 734 protein-GO term associations from Enrichr (35) to enrich the understanding of functional contexts.

For signature information, DrugRepoBank encompasses disease signature based on RNA-seq data from The Cancer Genome Atlas Program (TCGA) (36). Besides, drug signatures are represented by 473 647 replicate-consensus signatures obtained from the Level 5 L1000 dataset (GSE92742) (37), consisting of normalized moderated z-scores for 12 328 genes.

For literature information, the database collates 169 repositioned drugs derived from 134 valid literature sources, extracting critical details such as old and new targets, diseases, experimental evidence and supporting sentences from PubMed (38).

#### Processing flow of disease signature

In order to acquire the genetic characteristics of cancer diseases (disease signature) for drug repositioning predictions, the RNA-seq gene expression profile data from The Cancer Genome Atlas (TCGA) were acquired through the UCSC Xena Browser (https://xenabrowser.net/hub/). As depicted in Supplementary Table S2, cancer types containing only cancer samples without normal samples were excluded (11 cancer types), resulting in 25 cancer types with both cancer and normal samples. Differential expression analysis for each cancer type was conducted using the limma package (version 3.56.2) (39). Genes meeting the specified cutoff criteria (a minimum adjusted *P*-value of 0.05 and a fold change of 2) were identified as differentially expressed genes (DEGs) and employed as disease signatures in subsequent calculations.

### Prediction of potential repositioned drug candidates via multiple algorithms

**Figure 3.**
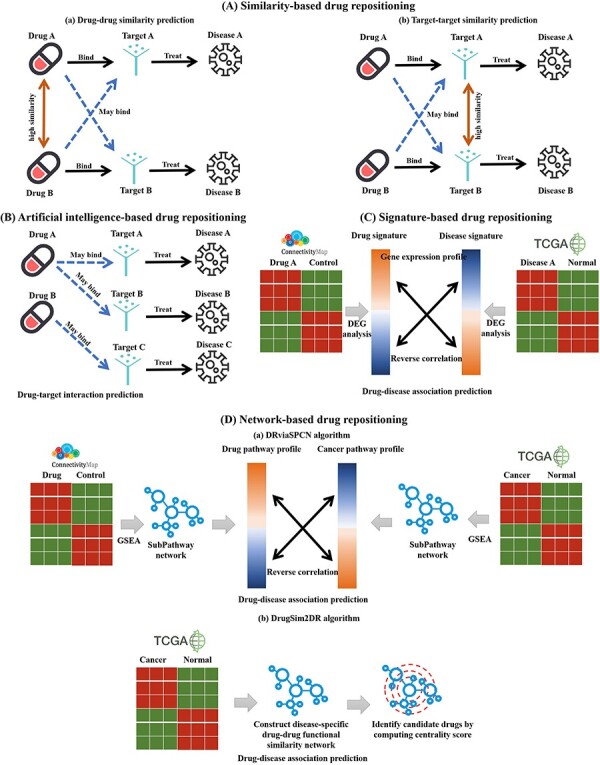
Schematic diagram of four kinds of drug repositioning algorithms integrated by DrugRepoBank. (A) Similarity-based drug repositioning. It contains two methods, namely (a) the drug–drug similarity prediction method and (b) the target–target similarity prediction method. (B) Artificial-intelligence-based drug repositioning. The approach is to predict drug–target interactions. (C) Signature-based drug repositioning. It operates on the fundamental premise that candidate drugs (Drug A) should reverse the gene signature (DEGs, Differentially Expressed Genes) associated with the disease of interest (Disease A), which has been altered by the disease when compared to control samples. (D) Network-based drug repositioning. (a) DRviaSPCN evaluates drug-disease reverse association based on disease- and drug-induced subpathways weighted by the subpathway crosstalk. (b) DrugSim2DR constructs a disease-specific functional drug–drug similarity network using gene expression data between cancer and normal states. Drugs are ranked based on network centrality scores calculated via a propagation algorithm.

The main purpose of drug repositioning is to detect new relationships between drugs and diseases or between drugs and targets. In the DrugRepoBank ‘Prediction’ module, we divided computational drug repositioning tools into four categories: similarity-based, artificial-intelligence-based, signature-based and network-based methods (Figure 3 and Table 3). For algorithm details, advantages and limitations on the sub-methods of each prediction method mentioned in the following text, please refer to Table S3 and the Supplementary Information.

**Table 3.** Drug repositioning methods integrated into DrugRepoBank

Methods class	Specific method	Detail	Ref
Similarity-based methods (drug–drug similarity prediction)	Chemical structure similarity	Chemical structure similarity is estimated with atom pairs using the Tanimoto coefficient, which is defined as the proportion of atom pairs shared among two compounds divided by their union.	(40)
	Target protein sequence-based similarity	Pairwise protein sequence comparison is performed using the standard Needleman-Wunsch dynamic programming algorithm for global alignment, and the percentage of pairwise sequence identity is reported as the corresponding sequence similarity	(41)
	Target Protein functional similarity [GO Cellular Component (CC)]	Each drug was annotated with enriched GO Cellular Component (CC) terms and the functional similarity between any two drugs is determined by the semantic similarity of their associated GO terms using the topology of the GO graph structure	(42)
	Target Protein functional similarity [GO Molecular Function (MF)]	Each drug was annotated with enriched GO Molecular Function (MF) terms and the functional similarity between any two drugs is determined by the semantic similarity of their associated GO terms using the topology of the GO graph structure	(42)
	Target Protein functional similarity [GO Biological Process (BP)]	Each drug was annotated with enriched GO Biological Process (BP) terms and the functional similarity between any two drugs is determined by the semantic similarity of their associated GO terms using the topology of the GO graph structure	(42)
	Drug-induced pathway similarity	Pairwise similarity between any two pathways was estimated based on the similarity of their constituent genes using dice similarity	(43)
Similarity-based method	Target-target similarity	Pairwise target protein sequences are compared based on the Needleman-Wunsch algorithm, which is designed based on dynamic programming	(41)
Artificial-intelligence-based methods	CPI_Prediciton	CPI_Prediciton is a CPI prediction approach by combining a graph neural network (GNN) for compounds and a convolutional neural network (CNN) for proteins	(44)
	TransformerCPI	TransformerCPI is a sequence-based deep learning method with a self-attention mechanism for compound-protein interaction prediction	(45)
	CapBM-DTI	CapBM-DTI is a drug-target interaction prediction method with capsule network and transfer learning	(46)
Signature-based methods	GSEAweight0	GSEAweight0 is derived from the KS-like statistic with weighted KS enrichment statistic (ES): p = 0	(47)
	GSEAweight1	GSEAweight1 is derived from the KS-like statistic with weighted KS enrichment statistic (ES): p = 1	(47)
	GSEAweight2	GSEAweight2 is derived from the KS-like statistic with weighted KS enrichment statistic (ES): p = 2	(47)
	KS	KS is derived from the KS-like statistic with the rank of fold changes as weight	(6)
	XSum	The XSum method was focused on the top genes ranked by fold changes of gene expression	(48)
	ZhangScore	The rank-based weights are set to all genes in one gene signature in ZhangScore	(49)
Network-based method	DRviaSPCN	DRviaSPCN is an approach to prioritize cancer candidate drugs by considering drug-induced subpathways and their crosstalk effects	(51)
	DrugSim2DR	DrugSim2DR is a tool that systematically predicts drug functional similarities within the context of specific diseases to facilitate drug repurposing	(52)

Similarity-based drug repositioning is based on the hypothesis that similar drugs tend to interact with similar targets and display similar therapeutic actions and, thus, can potentially treat a similar constellation of diseases. It contains two methods: the drug–drug similarity prediction method (Figure 3Aa) and the target–target similarity prediction method (Figure 3Ab). The drug–drug similarity prediction method calculates the similarity between the drug of interest and other drugs in multiple dimensions. For example, Drug A is more likely to bind with the target (Target B) of its most similar drug (Drug B) to treat a new disease (Disease B). The target–target similarity prediction method calculates the similarity between the target of interest and other targets. For instance, if the target (Target A) of Drug A is the most similar to Target B, then Drug A may be able to treat new diseases (Disease B) by combining the new target (Target B). As shown in Supplementary Figure S1A, the drug–drug similarity calculation process involves a comprehensive assessment of 49 652 small-molecule drugs from DrugBank, employing R packages to generate six distinct similarity matrices based on chemical structures (ChemmineR, version 3.54.0) (40), protein target sequences (Biostrings, version 2.70.3) (41), Gene Ontology annotations of Cellular Component, Biological Process, Molecular Function (GOSemSim, version 2.28.1) (42) and drug-induced pathways (BioCor, version 1.26.0) (43). These individual matrices are then mean-aggregated to create a combined-score similarity matrix, where ‘RowMeans’ represents the average similarity score across the six methods. Higher RowMeans values indicate a higher degree of overall similarity between drug pairs. Statistical significance is assessed through *P*-values (standardized z-scores) and adjusted *P*-values, where smaller values denote greater confidence in the similarity of drug pairs, with a lower false discovery rate. Pairs with missing values across all individual matrices, lacking SMILE structures, or consisting of non-marketed/approved drugs are excluded to ensure relevance. Supplementary Figure S1B illustrates the reliance of target–target similarity prediction on comparative analysis of protein sequences, facilitated by the Needleman-Wunsch algorithm, a dynamic programming technique. DrugRepoBank utilizes Biostrings R package (v2.70.3) (41) for estimation and excludes scores of ‘NA’ or less than 20 to obtain the final target–target similarity table.

As for the artificial-intelligence-based method, it extracts discriminative biological features for chemical compounds and target proteins in a drug–target pair and feeds the extracted features into an AI-based model such as random forest, logistic regression, convolution neural network (CNN), long short-term memory (LSTM) network to determine whether the drug and the target protein will interact or not. For example (Figure 3B), if Drug A can bind to new targets (Target A or Target B), it can further treat new diseases (Disease A or Disease B). As depicted in Supplementary Figure S2, we have integrated three AI-based methods [CPI_Prediction (https://github.com/masashitsubaki/CPI_prediction) (44), TransformerCPI (https://github.com/lifanchen-simm/transformerCPI) (45) and CapBM-DTI (https://github.com/huangyixian666/CapBM-DTI) (46)] within the DrugRepoBank to assess the predictive status and scores of drug–target interactions. The process begins by obtaining 49 652 drug SMILES structures and 4221 target protein sequences, which serve as inputs for each method. Each method assigns a binary ‘status’ (1 for association, 0 for no association) and generates a prediction score ranging from 0 to 1, with higher scores signifying a stronger likelihood of interaction. Two crucial aggregate metrics are then calculated: ‘Status mean’, the average status value across the three methods, and ‘Score mean’, the average prediction score. Finally, DrugRepoBank applies a filtering step to eliminate drug–target interactions with a ‘Status mean’ of 0, indicating that all three methods collectively predict no interaction. This rigorous evaluation and consolidation of results from multiple AI models yields a robust and refined drug–target interactions table.

The signature-based method has been widely used for screening drugs and identifying molecular actions of drugs, both in modern medicine and Traditional Chinese Medicine (TCM) [9, 10]. The hypothesis associated with this method involves the selection of a drug that has a reversal effect on the disease signature genes (Figure 3C). Briefly, suppose the pattern of the gene expression induced by a drug (drug signatures) contrasts that induced by a disease (disease signatures). In that case, the drug will demonstrate a therapeutic value for the disease. As shown in Supplementary Figure S3, we have incorporated six signature-based methods [GSEAweight0 (47), GSEAweight1 (47), GSEAweight2 (47), KS (6), XSum (48) and ZhangScore (49) via RCSM (50) R package (https://github.com/Jasonlinchina/RCSM)] into DrugRepoBank to provide a comprehensive assessment of disease–drug associations. Leveraging drug signatures (Level 5 data from L1000) and disease signatures (DEGs across 25 cancer types from TCGA) as inputs, these distinct methods independently compute the likelihood of a given drug effectively treating a specific disease. Subsequently, the ‘RowMeans’ are derived by standardizing the z-scores of the individual method outputs and averaging them. A lower ‘RowMeans’ value indicates a higher likelihood of the drug effectively treating cancer.

The network-based drug repositioning method harnesses integrated data from multiple sources, like pathway and drug similarity networks, to identify novel therapeutic uses for existing drugs in a holistic manner (51, 52). Recently, several network-based drug repositoning methods (51–55) have been proposed. SubtypeDrug (53) and DRviaSPCN (51) mainly assess drug-disease reverse correlations through gene expression or pathway activity levels, with DRviaSPCN being notable for incorporating crosstalk effects in cancer drug repositioning. DTSEA (54), PriorCD (55) and DrugSim2DR (52) all factor in drug similarities and interactions. DTSEA uses network propagation and enrichment analysis to repurpose drugs for COVID-19, PriorCD prioritizes cancer treatments via a pathway-focused similarity network and diffusion, while DrugSim2DR uniquely considers the incorporation of molecular characteristics within the context of a specific disease state to infer drug repositioning possibilities. We chose DRviaSPCN version 0.1.4 (Figure 3D(a)) and DrugSim2DR version 0.1.1 (Figure 3D(b)) for drug repositioning within DrugRepoBank due to their ability to handle subpathway crosstalk and disease-specific molecular properties. As shown in Supplementary Figure S4, both DRviaSPCN and DrugSim2DR algorithms incorporate disease signatures (DEGs across 25 cancer type from TCGA) to generate potential drug tables. DRviaSPCN computes subpathway Centrality Scores, reflecting crosstalk impact in the context of input cancer signatures, and uses these scores to derive drug enrichment scores (DES) for a repositioning candidate table. Smaller DES indicate a higher chance of a drug’s effectiveness against cancer, with statistical significance determined by the False Discovery Rate (FDR); lower *P*-values denote higher confidence. Conversely, DrugSim2DR computes drug centrality scores based on cancer signatures, ultimately yielding a separate table of potential drug repositioning candidates. Here, higher centrality scores suggest a stronger likelihood that a drug treats a particular disease, and the FDR is also employed to gauge reliability, with lower FDR values representing increased confidence in the results.

### Technical background

The data of DrugRepoBank are stored in a MySQL database (version 15.1) on a LAMP (Linux/Apache/MySQL/PHP) server (CentOS Linux release 7.9.2009). The entire database website is built using PHP (version 7.4.33) as the backend language to connect to the MySQL database to query data from it and provide the queried data to the front end. The front end of the website uses HTML5 to construct webpages, adjust the appearance and style through CSS and provide interactive functionality via JavaScript and jQuery library (version 3.6.0, https://jquery.com/). Smarty (version 3.1.30, https://www.smarty.net/) is used as a template engine for PHP to facilitate a manageable way to separate application logic and content from its presentation. Bootstrap (version 3.4.1, https://getbootstrap.com/), as a basic framework, provides the foundation for website construction, and the website utilizes its various functions. The network is created with the Force Directed Tree in JavaScript library amCharts 4 (version 4.10.36, https://www.amcharts.com/). Tables with sorting and search functions are constructed by the JSTable JavaScript plugin (version 1.6.5, https://jstable.github.io/). Data processing is usually done through Python (version 3.9.6, https://www.python.org/) and R (version 4.2.2, https://www.r-project.org/) software. This website can be used generally on Google Chrome, Microsoft Edge, etc.

## Results

### Analysis of literature-supported drug repositioning data

The ‘Literature’ module is a manual collection of experimentally validated drug repositioning information. Our database contains 169 repositioned drugs from 134 papers (Figure 2B). To systematically analyze the experimental methods used in the published literature on repositioning drugs, we display the number of repositioning drugs at various experimental stages in a Venn diagram (Figure 2C, the overlapping sections show the drugs that fall into multiple evidence categories, highlighting their multi-level evidence support) and the frequency distribution of each evidence in all four experiment levels in Pie chart (Figure 2D), which illustrates the diversity of detail experiment evidence for the repositioning drugs. In Figure 2C, *in vitro* experiments comprise the largest proportion (131 repositioned drugs), and clinical trials represent the smallest (5 repositioned drugs), which reveals the universality of *in vitro* experimental evidence and the scarcity of clinical research in the scientific research of drug repositioning. No repositioning drug was verified by four experimental methods simultaneously, while five repositioning drugs were verified by three experimental methods other than clinical research. Most repositioning drugs are verified by two experimental methods, especially the combination of *in silico* and *in vitro* experiments (21 repositioned drugs) and the combination of *in vitro* and *in vivo* experiments (24 repositioned drugs). Figure 2D shows the proportion of experimental approaches for the four types of experimental evidence. *In vitro* experimental evidence (red section) is mainly composed of Western Blot (40 repositioned drugs, 16%) and RT-qPCR (23 repositioned drugs, 9%), while molecular docking (14 repositioned drugs, 6%) and molecular dynamic simulation (12 repositioned drugs, 5%) occupy a major position in *in silico* experimental evidence (blue section). As for *in vivo* experiments (green section), mouse modeling (13 repositioned drugs, 5%) and xenograft (10 repositioned drugs, 4%) are the most commonly used methods. However, limited literature (five repositioned drugs, 2%) has mentioned clinical trials (yellow section) with the highest evidence level.

### Characteristics and mechanisms of potential candidate drugs and targets identified through multi-algorithmic prediction

The merged result of high-confidence (*P* < 0.05) drug–drug similarities predicted by six signature-based drug repositioning methods comprises 238 635 pairs. The individual method contributions are as follows: 2175 pairs for chemical structure similarity, 33 226 pairs for target protein sequence-based similarity, 223 699 pairs for target protein functional similarity based on GO Cellular Component (CC), 235 622 pairs for target protein functional similarity based on GO Molecular Function (MF), 225 027 pairs for target protein functional similarity based on GO Biological Process (BP) and 39 164 pairs for drug-induced pathway similarity. The intersection of drug–drug similarities predicted by the six methods is detailed in Supplementary Figure S5. Besides, there are 4 436 407 pairs of high-confidence (similarity score ≥20) target–target similarity.

The results of AI-based drug repositioning are depicted in Supplementary Figure S6, which shows that the three employed methods, namely CPI_Prediction, TransformerCPI and CapBM-DTI, yield respective predictions of 3 940 141, 24 236 758 and 29 817 392 drug–target interactions. The intersection among these predictions consists of 1 746 583 high-confidence drug–target interactions.

The prediction results for drug repositioning candidates across 25 cancer types are detailed in Supplementary Table S4, which tabulates the Mechanisms of Action (MOA) categories (occurring twice or more) and corresponding specific drugs within the Top 50 repositioning candidates identified by six signature-based methods and two network-based methods, namely DRviaSPCN and DrugSim2DR. The six signature-based methods predicted that histone deacetylase (HDAC) inhibitors would be significantly represented among cancer therapies, with 13 occurrences. Similarly, MEK (Mitogen-Activated Protein Kinase/Extracellular Signal-Regulated Kinase (MAPK/ERK) Kinase) inhibitors and topoisomerase inhibitors were also anticipated, each with nine occurrences noted. Regarding DRviaSPCN, it was observed that antibiotics, antihypertensive agents and antipsychotic agents would be prominent in cancer therapy, with respective occurrences of 13, 10 and 6. In the context of DrugSim2DR, antihypertensive agents, analgesics and antipsychotic agents were each predicted to play a role in cancer treatment, with 8, 6 and 6 occurrences, respectively.

### Comparison between DrugRepoBank and other databases

Compared to existing drug repositioning databases, DrugRepoBank distinguishes itself as a comprehensive resource with multi-data, multi-functionality, multiple algorithms and a user-friendly interface. Other drug repositioning databases have their unique characteristics and areas of focus. For example, DrugSimDB primarily emphasizes similarity-based methods, NeDRex specializes in network-based approaches and Connectivity Map, DrugSig, LINCS Data Portal 2.0 and DREIMT concentrate on signature analysis. Promiscuous, Promiscuous 2.0 and PharmOmics employ dual algorithms for predictions. In contrast, RepurposeDB and repoDB lack prediction capabilities, and EK-DRD specializes in collecting literature data for repurposed drugs.

DrugRepoBank exhibits distinct advantages regarding data volume and diversity, predictive algorithms, functional analysis and user-friendliness. Firstly, regarding the data, in contrast to numerous existing drug repositioning databases that specialize in specific data types aligned with their primary objectives, DrugRepoBank provides a comprehensive range of extensive and diverse information, encompassing drugs, targets, side effects, diseases, pathways and their interactions or associations. Importantly, DrugRepoBank meticulously curates literature from January 2000 to July 2023. Secondly, to uncover potential drug repositioning candidates, DrugRepoBank integrates multiple advanced approaches, including seven similarity-based algorithms, three artificial intelligence-based algorithms, six signature-based algorithms and two network-based algorithms. Thirdly, DrugRepoBank integrates a bioinformatics analysis pipeline that encompasses differential gene expression analysis, GO and KEGG functional analysis and subpathway functional analysis, which provides valuable insights into the biological functions and metabolic processes that diseases can influence. Lastly, DrugRepoBank features an interactive and user-friendly web interface. This interface facilitates easy access to the database, search, filtering and data export. It also incorporates a network visualization module to clarify the interactions and associations among drugs, targets, diseases and side effects.

### User-friendly web interface

**Figure 4.**
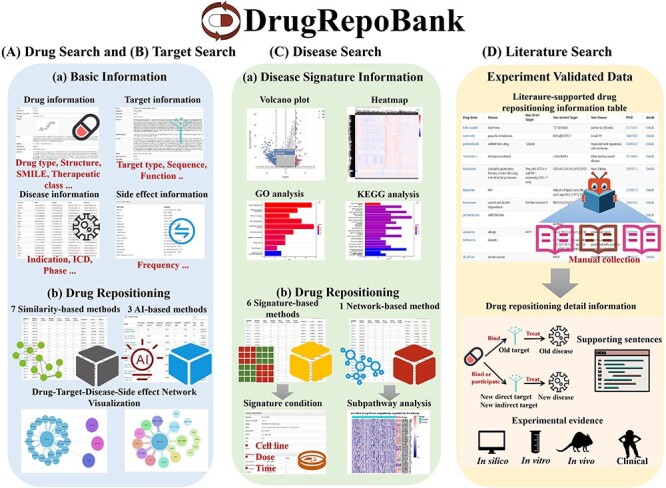
Demonstration of web interfaces. There are two main modules: the ‘Prediction’ module and the ‘Literature’ module. The ‘Prediction’ module consists of (A) Drug Search, (B) Target Search and (C) Disease Search. The ‘Literature’ module has a (D) literature search engine.

Figure 4 and Supplementary Figure S8 show that DrugRepoBank has a user-friendly web interface for data presentation, search and visualization. There are two main modules in DrugRepoBank: the ‘Prediction’ module and the ‘Literature’ module. The ‘Prediction’ module aims to achieve the goal of drug repositioning by identifying new relationships between drugs and diseases or drugs and targets through multiple algorithms based on three search engines [Drug Search (Figure 4A), Target Search (Figure 4B) and Disease Search (Figure 4C)]. The Drug Search and Target Search provide basic information about the drug (drug type, structure, SMILE, therapeutic class, etc.), target (target type, sequence, function, etc.), disease (indication, ICD, phase, etc.) and side effect (frequency), as well as drug repositioning which includes the drug repositioning scoring table predicted through multiple algorithms, including seven similarity-based methods and three artificial intelligence-based methods, along with drug-target-disease-side effect network visualization. Disease Search provides disease signature information, including DEG analysis in a volcano plot, heatmap and pathway analysis encompassing KEGG and GO analysis of up-regulated genes, down-regulated genes and a combination of DEGs. Drug repositioning also includes six signature-based methods offering signature conditions and two network-based methods providing subpathway analysis. The ‘Literature’ module is an experimentally validated drug repositioning part through a manual curation approach from the PubMed database, intending to provide literature support for existing repositioning drugs and uncover patterns in the discovery of repurposed drugs. Literature Search provides an experimentally validated drug repositioning table, including information such as old drug, old disease, new direct target, new indirect target, new disease, experiment evidence (*in silico, in vitro, in vivo* and clinical trials), supporting sentences and PMID (PubMed ID).

For detailed information on the web interface, please refer to the Supplementary Information.

### Case studies to demonstrate the accuracy, reliabilty and feasibility of predictively repositioned drugs and targets within DrugRepoBank

To further demonstrate the practical relevance and experimental validity of DrugRepoBank’s predictive algorithms in identifying potential candidate drugs and targets, we delve into three illustrative examples. These case studies showcase the successful application of our platform in diverse disease contexts, highlighting the alignment between DrugRepoBank’s predictions and established experimental evidence from the scientific literature. Through these real-world scenarios, we provide concrete demonstrations of how DrugRepoBank’s predictive capabilities can effectively reveal promising drug repositioning opportunities with a solid foundation in empirical data, thereby reinforcing the platform’s utility in fostering translational research and guiding therapeutic strategies.

#### Case Study 1: Literature-supported experimental validation of dabigatran as a confirmed oral alternative for heparin-induced thrombocytopenia via similarity-based drug repositioning

**Figure 5.**
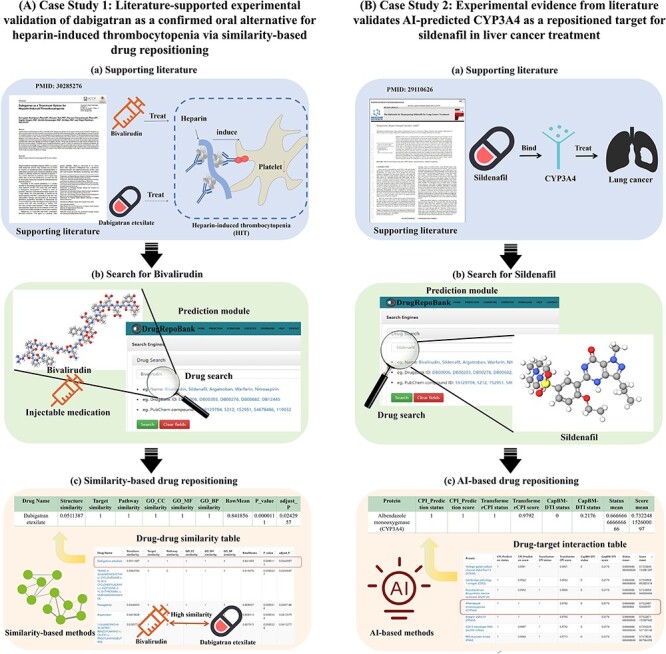
Case studies 1 and 2 demonstrate the accuracy, reliability and feasibility of predictively repositioned drugs within DrugRepoBank. (A) Case Study 1: Literature-supported experimental validation of dabigatran as a confirmed oral alternative for heparin-induced thrombocytopenia via similarity-based drug repositioning. (a) Literature supports that Dabigatran is a possible candidate for oral anticoagulant for treating HIT. (b) Bivalirudin is a commonly injectable medication to treat HIT. Use the ‘Prediction’ module of DrugRepoBank by typing ‘Bivalirudin’ in the drug search box and clicking ‘Search’. (c) Sort the results by the ‘RowMean’ and find that dabigatran etexilate has a high similarity with bivalirudin (row means = 0.841856, ranking = first). (B) Case study 2: Experimental evidence from literature validates AI-predicted CYP3A4 as a repositioned target for sildenafil in liver cancer treatment. (a) Literature confirmed that sildenafil can potentially be used in treating lung cancer by targeting CYP3A4. (b) Drug search for sildenafil, which is used to treat penile erectile dysfunction. (c) We conducted the ‘AI-based Drug Repositioning’ for sildenafil, sorted the result by ‘Score mean’ and identified CYP3A4 as potential targets (score mean = 0.732, ranking = fourth).

Heparin-induced thrombocytopenia (HIT) is a severe immune-mediated response that substantially amplifies the risk of clotting complications within both arteries and veins. To manage HIT, injectable medications like bivalirudin are commonly employed. Aligning with the 2018 American Society of Hematology (ASH) Guideline, incorporating oral anticoagulants is suggested to optimize cost-effectiveness and procedural simplicity for HIT (56). A recent study (57) has demonstrated that Dabigatran could be considered a safe and effective agent in the management of HIT by selectively binding to both free and clot-bound thrombin via clinical trial of 43 patients. Hence, we employed DrugRepoBank to investigate the similarity between dabigatran and bivalirudin as a case study, providing evidence that DrugRepoBank can identify similar drugs for disease treatment through similarity-based drug repositioning (Figure 5Aa). Consequently, we explore bivalirudin in the ‘Drug Search’ of the ‘Prediction’ module (Figure 5Ab). By the drug–drug similarity table presented in the ‘Similarity-based drug repositioning’ module (Figure 5Ac), it is noteworthy that dabigatran etexilate exhibits high similarity scores (row means = 0.841856, ranking = first) when compared with Bivalirubin, which validates the effectiveness of similarity-based drug repositioning.

#### Case Study 2: Experimental evidence from literature validates AI-predicted CYP3A4 as a repositioned target for sildenafil in liver cancer treatment

Sildenafil is a medication for the treatment of penile erectile dysfunction by inhibiting guanosine monophosphate in the corpus cavernosum (58). Current research demonstrates that sildenafil may serve as a potential agent for the treatment of lung cancer by inhibiting CYP3A4 through *in vitro* enzymatic assays, drug interaction investigations and pharmacokinetic studies (59). To demonstrate the effectiveness of AI-based methods in identifying new drug targets for treating new diseases, we investigated whether sildenafil could bind to CYP3A4 and thus serve as a potential treatment for liver cancer as a case study (Figure 5 Ba). Therefore, we inputted sildenafil into the ‘Drug Search’ of the ‘Prediction module’ (Figure 5Bb). As demonstrated in Figure 5Bc, we sorted the results of ‘AI-based drug repositioning’ by ‘Score mean’ and identified CYP3A4 as a potential target protein with a relatively high score (score mean = 0.732, ranking = fourth), showing that the AI-based methods can effectively predict new drug targets, thereby facilitating drug repositioning.

#### Case Study 3: Literature-confirmed efficacy of panobinostat and verteporfin as novel lung cancer therapeutics via experimental validation of signature- and network-based drug repositioning

**Figure 6.**
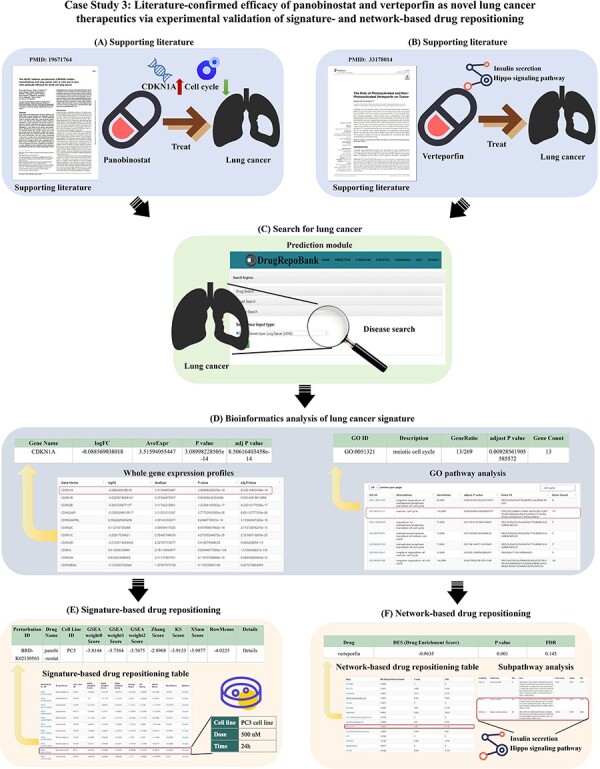
Case study 3: Literature-confirmed efficacy of panobinostat and verteporfin as novel lung cancer therapeutics via experimental validation of signature- and network-based drug repositioning. (A) Literature shows that panobinostat is a potential drug for lung cancer treatment affecting both the cell cycle and CDKN1A. (B) Verteporfin has been demonstrated in previous studies to effectively restrain the proliferation and migration of lung cancer cells by modulating the Hippo signaling pathway and insulin secretion. (C) ‘Disease Search’ for lung cancer. (D) The bioinformatics analysis reveals CDKN1A, a cell cycle regulator, is up-regulated, and the cell cycle pathway is enhanced in lung cancer patients. (E) Based on signature-based drug repositioning, we sorted the results by ‘RowMean’ and found panobinostat with a relatively high score (Rowmean = –3.4225, ranking = 30th). (F) Based on the network-based drug repositioning, we sorted the results by DES and found verteporfin with a relatively low score (DES = –0.9635, ranking = seventh). Besides, the ‘Subpathway Analysis’ found two possible subpathways for lung cancer: the Hippo signaling pathway and insulin secretion.

Lung cancer ranks as the third most prevalent form of cancer and stands as the second leading cause of cancer-related fatalities (60). Therefore, it is essential to explore drug repositioning strategies for treating lung cancer. Panobinostat exhibited a marked reduction in tumor growth, accompanied by inhibition of the cell cycle pathway and a decrease in cell cycle regulators like CDKN1A via *in vitro* cytotoxicity assays and *in vivo* xenograft models (Figure 6A) (61). Verteporfin has been demonstrated to effectively restrain the proliferation and migration of lung cancer cells through the modulation of the Hippo signaling pathway and insulin secretion with the experimental evidence of *in vitro* cytotoxicity and apoptosis assays and *in vivo* xenograft models (Figure 6B) (62). To discover new drugs for treating diseases through signature-based and network-based drug repositioning, we explored the effectiveness of these two algorithms using the above two case studies in DrugRepoBank.

We undertook a drug repositioning study for lung cancer utilizing our DrugRepoBank database by searching ‘LUNG’ in the ‘Disease Search’ of the ‘Prediction’ module (Figure 6C). Through the bioinformatics analysis (DEG analysis and GO and KEGG pathway enrichment analysis) of disease signature (Figure 6D), we found that CDKN1A, a cell cycle regulator, is up-regulated, and the cell cycle pathway is enhanced in lung cancer patients. Employing both signature-based (Figure 6E) and network-based approaches (Figure 6F) within our database, we identified two potential candidates, panobinostat (Rowmean = –3.4225, ranking = 30th) and verteporfin (DES = –0.9635, ranking = seventh), for the treatment of lung cancer. With bioinformatics analysis, it can be inferred that panobinostat inhibits liver cancer growth by suppressing the cell cycle pathway and downregulating CDKN1A, which aligns with reported literature (61). Subpathway analysis indicates that verteporfin may influence lung cancer by modulating the Hippo signaling pathway and insulin secretion, consistent with literature findings (62).

## Conclusion and discussion

In conclusion, DrugRepoBank constitutes a pioneering answer to the pressing demands in the domain of drug repositioning, surmounting prevalent challenges such as inadequate data availability, algorithmic inaccuracies, deficient literature integration and user-unfriendly interfaces common among existing databases. DrugRepoBank offers a comprehensive and innovative solution by aggregating vast amounts of diverse data from high-quality sources, incorporating manually extracted, experimentally validated repositioning cases and deploying advanced multi-dimensional algorithms. It also provides a suite of analytical tools that enable in-depth exploration through network visualizations and pathway analyses within a highly user-friendly interface.

Looking ahead, we are dedicated to continuously updating and enriching our database to encompass more repositioned drugs, integrating multi-omics data for both drugs and diseases, refining our predictive algorithms to achieve greater accuracy, and consistently emphasizing the paramount importance of experimental validation. DrugRepoBank will accelerate drug repositioning for pharmacists by pinpointing potential drugs, help biologists discover new mechanisms and targets and empower computational biologists to refine algorithms. To maximize the benefits of drug repositioning, we urge global stakeholders to establish stringent monitoring and surveillance mechanisms to guarantee the long-term safety of repositioned drugs, and concurrently, to formulate corresponding policies and regulations protecting intellectual property rights for repurposed medications. Ultimately, we wish DrugRepoBank as a valuable resource that not only fosters the discovery of new drug indications but also expedites their practical implementation in the clinic, thereby revolutionizing the drug repositioning landscape.

## Supplementary Material

baae051_Supp

## Data Availability

DrugRepoBank is publicly accessible at https://awi.cuhk.edu.cn/DrugRepoBank.

## References

[1] Ashburn T.T. and Thor K.B. (2004) Drug repositioning: identifying and developing new uses for existing drugs. *Nat. Rev. Drug Discov*., 3, 673–683. 15286734 10.1038/nrd1468

[2] Olgen S. (2019) A prospective overview of drug repurposing in drug discovery and development. *Curr. Med. Chem*., 26, 5338–5339. 31721690 10.2174/092986732628191025094454

[3] Chong C.R. and Sullivan D.J. Jr (2007) New uses for old drugs. *Nature*, 448, 645–646. 17687303 10.1038/448645a

[4] Xue H. , Li J., Xie H. et al. (2018) Review of drug repositioning approaches and resources. *Int. J. Biol. Sci*., 14, 1232–1244. 30123072 10.7150/ijbs.24612 PMC6097480

[5] Aggarwal S. , Verma S.S., Aggarwal S. et al. (2021) Drug repurposing for breast cancer therapy: old weapon for new battle. *Semin. Cancer Biol*., 68, 8–20. 31550502 10.1016/j.semcancer.2019.09.012 PMC7128772

[6] Lamb J. , Crawford E.D., Peck D. et al. (2006) The connectivity map: using gene-expression signatures to connect small molecules, genes, and disease. *Science*, 313, 1929–1935. 17008526 10.1126/science.1132939

[7] Von Eichborn J. , Murgueitio M.S., Dunkel M. et al. (2010) PROMISCUOUS: a database for network-based drug-repositioning. *Nucleic Acids Res*., 39, D1060–D1066. 21071407 10.1093/nar/gkq1037 PMC3013657

[8] Wu H. , Huang J., Zhong Y. et al. (2017) DrugSig: a resource for computational drug repositioning utilizing gene expression signatures. *PLoS One*, 12, e0177743. 10.1371/journal.pone.0177743 PMC5451001 28562632

[9] Brown A.S. and Patel C.J. (2017) A standard database for drug repositioning. *Sci. Data*, 4, 170029. 10.1038/sdata.2017.29 PMC5349249 28291243

[10] Shameer K. , Glicksberg B.S., Hodos R. et al. (2018) Systematic analyses of drugs and disease indications in RepurposeDB reveal pharmacological, biological and epidemiological factors influencing drug repositioning. *Brief Bioinform*., 19, 656–678. 28200013 10.1093/bib/bbw136 PMC6192146

[11] Zhao C. , Dai X., Li Y. et al. (2019) EK-DRD: a comprehensive database for drug repositioning inspired by experimental knowledge. *J. Chem. Inf. Model*., 59, 3619–3624. 31433187 10.1021/acs.jcim.9b00365

[12] Gallo K. , Goede A., Eckert A. et al. (2021) PROMISCUOUS 2.0: a resource for drug-repositioning. *Nucleic Acids Res*., 49, D1373–D1380. 33196798 10.1093/nar/gkaa1061 PMC7779026

[13] Stathias V. , Turner J., Koleti A. et al. (2020) LINCS data portal 2.0: next generation access point for perturbation-response signatures. *Nucleic Acids Res*., 48, D431–d439. 31701147 10.1093/nar/gkz1023 PMC7145650

[14] Sadegh S. , Skelton J., Anastasi E. et al. (2021) Network medicine for disease module identification and drug repurposing with the NeDRex platform. *Nat. Commun*., 12, 6848. 10.1038/s41467-021-27138-2 PMC8617287 34824199

[15] Azad A.K.M. , Dinarvand M., Nematollahi A. et al. (2021) A comprehensive integrated drug similarity resource for in-silico drug repositioning and beyond. *Brief Bioinform*., 22, bbaa126. 10.1093/bib/bbaa126 32597467

[16] Troulé K. , López-Fernández H., García-Martín S. et al. (2021) DREIMT: a drug repositioning database and prioritization tool for immunomodulation. *Bioinformatics*, 37, 578–579. 32818254 10.1093/bioinformatics/btaa727

[17] Chen Y.W. , Diamante G., Ding J. et al. (2022) PharmOmics: a species- and tissue-specific drug signature database and gene-network-based drug repositioning tool. *iScience*, 25, 104052. 10.1016/j.isci.2022.104052 PMC8957031 35345455

[18] Wishart D.S. , Feunang Y.D., Guo A.C. et al. (2018) DrugBank 5.0: a major update to the DrugBank database for 2018. *Nucleic Acids Res*., 46, D1074–D1082. 29126136 10.1093/nar/gkx1037 PMC5753335

[19] Kim S. , Chen J., Cheng T. et al. (2023) PubChem 2023 update. *Nucleic Acids Res*., 51, D1373–D1380. 36305812 10.1093/nar/gkac956 PMC9825602

[20] Zhou Y. , Zhang Y., Lian X. et al. (2022) Therapeutic target database update 2022: facilitating drug discovery with enriched comparative data of targeted agents. *Nucleic Acids Res*., 50, D1398–d1407. 34718717 10.1093/nar/gkab953 PMC8728281

[21] Zhou Y. , Zhang Y., Zhao D. et al. (2024) TTD: Therapeutic Target Database describing target druggability information. *Nucleic Acids Res*., 52, D1465–D1477. 37713619 10.1093/nar/gkad751 PMC10767903

[22] Kuhn M. , Letunic I., Jensen L.J. et al. (2016) The SIDER database of drugs and side effects. *Nucleic Acids Res*., 44, D1075–9. 26481350 10.1093/nar/gkv1075 PMC4702794

[23] Kanehisa M. , Furumichi M., Sato Y. et al. (2021) KEGG: integrating viruses and cellular organisms. *Nucleic Acids Res*., 49, D545–d551. 33125081 10.1093/nar/gkaa970 PMC7779016

[24] Li F. , Yin J., Lu M. et al. (2023) DrugMAP: molecular atlas and pharma-information of all drugs. *Nucleic Acids Res*., 51, D1288–D1299. 36243961 10.1093/nar/gkac813 PMC9825453

[25] Sun X. , Zhang Y., Li H. et al. (2023) DRESIS: the first comprehensive landscape of drug resistance information. *Nucleic Acids Res*., 51, D1263–D1275. 36243960 10.1093/nar/gkac812 PMC9825618

[26] Zhang Y. , Zhou Y., Zhou Y. et al. (2024) TheMarker: a comprehensive database of therapeutic biomarkers. *Nucleic Acids Res*., 52, D1450–D1464. 37850638 10.1093/nar/gkad862 PMC10767989

[27] Yin J. , Chen Z., You N. et al. (2024) VARIDT 3.0: the phenotypic and regulatory variability of drug transporter. *Nucleic Acids Res*., 52, D1490–D1502. 37819041 10.1093/nar/gkad818 PMC10767864

[28] Zhang Y. , Liu X., Li F. et al. (2024) INTEDE 2.0: the metabolic roadmap of drugs. *Nucleic Acids Res*., 52, D1355–D1364. 37930837 10.1093/nar/gkad1013 PMC10767827

[29] Burley S.K. , Berman H.M., Kleywegt G.J. et al. (2017) Protein Data Bank (PDB): the single global macromolecular structure archive. *Protein Crystallogr*. 1607, 627–641. 10.1007/978-1-4939-7000-1_26 PMC5823500 28573592

[30] Martens M. , Ammar A., Riutta A. et al. (2021) WikiPathways: connecting communities. *Nucleic Acids Res*., 49, D613–d621. 33211851 10.1093/nar/gkaa1024 PMC7779061

[31] Pon A. , Jewison T., Su Y. et al. (2015) Pathways with PathWhiz. *Nucleic Acids Res*., 43, W552–9. 25934797 10.1093/nar/gkv399 PMC4489271

[32] Jassal B. , Matthews L., Viteri G. et al. (2020) The reactome pathway knowledgebase. *Nucleic Acids Res*., 48, D498–d503. 31691815 10.1093/nar/gkz1031 PMC7145712

[33] Kandasamy K. , Mohan S.S., Raju R. et al. (2010) NetPath: a public resource of curated signal transduction pathways. *Genome Biol*., 11, R3. 10.1186/gb-2010-11-1-r3 PMC2847715 20067622

[34] Mi H. , Muruganujan A., Ebert D. et al. (2019) PANTHER version 14: more genomes, a new PANTHER GO-slim and improvements in enrichment analysis tools. *Nucleic Acids Res*., 47, D419–D426. 30407594 10.1093/nar/gky1038 PMC6323939

[35] Xie Z. , Bailey A., Kuleshov M.V. et al. (2021) Gene set knowledge discovery with Enrichr. *Curr. Protoc*., 1, e90. 10.1002/cpz1.90 PMC8152575 33780170

[36] Tomczak K. , Czerwińska P. and Wiznerowicz M. (2015) The Cancer Genome Atlas (TCGA): an immeasurable source of knowledge. *Contemp. Oncol. (Pozn)*, 19, A68–77. 25691825 10.5114/wo.2014.47136 PMC4322527

[37] Subramanian A. , Narayan R., Corsello S.M. et al. (2017) A next generation connectivity map: L1000 Platform and the First 1,000,000 Profiles. *Cell*, 171, 1437–1452.e17. 29195078 10.1016/j.cell.2017.10.049 PMC5990023

[38] Canese K. Weis S. (2013) PubMed: the bibliographic database. In: Beck J, Benson D and Coleman J et al. (eds.) *The NCBI Handbook*, Vol. 2. Bethesda, MD, USA: the National Center for Biotechnology Information (NCBI), 1–61.

[39] Smyth G.K. (2005) *Limma: Linear Models for Microarray Data. Bioinformatics and Computational Biology Solutions Using R and Bioconductor*. New York, NY, USA, Springer, 397–420.

[40] Cao Y. , Charisi A., Cheng L.-C. et al. (2008) ChemmineR: a compound mining framework for R. *Bioinformatics*, 24, 1733–1734. 18596077 10.1093/bioinformatics/btn307 PMC2638865

[41] Pagès H. , Aboyoun P., Gentleman R. et al. (2019) Biostrings: efficient manipulation of biological strings. R Package Version 2 10.18129.

[42] Yu G. , Li F., Qin Y. et al. (2010) GOSemSim: an R package for measuring semantic similarity among GO terms and gene products. *Bioinformatics*, 26, 976–978. 20179076 10.1093/bioinformatics/btq064

[43] Sancho L. (2019) BioCor: functional similarities. R package version.

[44] Tsubaki M. , Tomii K. and Sese J. (2019) Compound–protein interaction prediction with end-to-end learning of neural networks for graphs and sequences. *Bioinformatics*, 35, 309–318. 29982330 10.1093/bioinformatics/bty535

[45] Chen L. , Tan X., Wang D. et al. (2020) TransformerCPI: improving compound–protein interaction prediction by sequence-based deep learning with self-attention mechanism and label reversal experiments. *Bioinformatics*, 36, 4406–4414. 32428219 10.1093/bioinformatics/btaa524

[46] Huang Y. , Huang H.-Y., Chen Y. et al. (2023) A robust drug–target interaction prediction framework with capsule network and transfer learning. *Int. J. Mol. Sci*., 24, 14061. 10.3390/ijms241814061 PMC10531393 37762364

[47] Subramanian A. , Tamayo P., Mootha V.K. et al. (2005) Gene set enrichment analysis: a knowledge-based approach for interpreting genome-wide expression profiles. *Proc. Natl. Acad. Sci*., 102, 15545–15550. 16199517 10.1073/pnas.0506580102 PMC1239896

[48] Cheng J. , Yang L., Kumar V. et al. (2014) Systematic evaluation of connectivity map for disease indications. *Genome Med*., 6, 1–8. 25606058 10.1186/s13073-014-0095-1 PMC4278345

[49] Zhang S.-D. and Gant T.W. (2008) A simple and robust method for connecting small-molecule drugs using gene-expression signatures. *BMC Bioinf*., 9, 1–10. 10.1186/1471-2105-9-258 PMC2464610 18518950

[50] Lin K. , Li L., Dai Y. et al. (2020) A comprehensive evaluation of connectivity methods for L1000 data. *Briefings Bioinf*., 21, 2194–2205. 10.1093/bib/bbz129 31774912

[51] Wu J. , Li X., Wang Q. et al. (2022) DRviaSPCN: a software package for drug repurposing in cancer via a subpathway crosstalk network. *Bioinformatics*, 38, 4975–4977. 36066432 10.1093/bioinformatics/btac611

[52] Wu J. , Li J., He Y. et al. (2023) DrugSim2DR: systematic prediction of drug functional similarities in the context of specific disease for drug repurposing. *GigaScience*, 12, giad104. 10.1093/gigascience/giad104 PMC10729734 38116825

[53] Han X. , Kong Q., Liu C. et al. (2021) SubtypeDrug: a software package for prioritization of candidate cancer subtype-specific drugs. *Bioinformatics*, 37, 2491–2493. 33459772 10.1093/bioinformatics/btab011

[54] Su Y. , Wu J., Li X. et al. (2023) DTSEA: a network-based drug target set enrichment analysis method for drug repurposing against COVID-19. *Comput. Biol. Med*., 159, 106969. 10.1016/j.compbiomed.2023.106969 PMC10121077 37105108

[55] Di J. , Zheng B., Kong Q. et al. (2019) Prioritization of candidate cancer drugs based on a drug functional similarity network constructed by integrating pathway activities and drug activities. *Mol. Oncol*., 13, 2259–2277. 31408580 10.1002/1878-0261.12564 PMC6763777

[56] Cuker A. , Arepally G.M., Chong B.H. et al. (2018) American Society of Hematology 2018 guidelines for management of venous thromboembolism: heparin-induced thrombocytopenia. *Blood Adv*., 2, 3360–3392. 30482768 10.1182/bloodadvances.2018024489 PMC6258919

[57] Nasiripour S. , Saif M., Farasatinasab M. et al. (2019) Dabigatran as a treatment option for heparin-induced thrombocytopenia. *J. Clin. Pharmacol*., 59, 107–111. 30285276 10.1002/jcph.1300

[58] Moreland R.B. , Goldstein I. and Traish A. (1998) Sildenafil, a novel inhibitor of phosphodiesterase type 5 in human corpus cavernosum smooth muscle cells. *Life Sci*., 62, L309–PL318. 10.1016/s0024-3205(98)00158-1 9600334

[59] Keats T. , Rosengren R.J. and Ashton J.C. (2018) The rationale for repurposing sildenafil for lung cancer treatment. *Anti Cancer Agents Med. Chem*., 18, 367–374. 10.2174/1871520617666171103100959 29110626

[60] Bade B.C. and Dela Cruz C.S. (2020) Lung cancer 2020: epidemiology, etiology, and prevention. *Clinics Chest Med*., 41, 1–24. 10.1016/j.ccm.2019.10.001 32008623

[61] Crisanti M.C. , Wallace A.F., Kapoor V. et al. (2009) The HDAC inhibitor panobinostat (LBH589) inhibits mesothelioma and lung cancer cells in vitro and in vivo with particular efficacy for small cell lung cancer. *Mol. Cancer Ther*., 8, 2221–2231. 19671764 10.1158/1535-7163.MCT-09-0138 PMC3605895

[62] Wei C. and Li X. (2020) The role of photoactivated and non-photoactivated verteporfin on tumor. *Front. Pharmacol*., 11. 10.3389/fphar.2020.557429 PMC7593515 33178014

