# Supplementary material for: DrugRepoBank: a comprehensive database and discovery platform for accelerating drug repositioning
Source: Database (Oxford). 2024 Jul 11;2024:baae051. doi: 10.1093/database/baae051 (PMC11240114; doi:10.1093/database/baae051)
Supplement: baae051_Supp [file baae051_supp.zip › suppl_data/Supplementary-CLEAN.docx]

**Table S1. Clinical status data statistics for drugs in DrugRepoBank**

| **Clinical status** | **Number** | **Clinical status** | **Number** |
| --- | --- | --- | --- |
| Investigative | 19573 | Preregistration | 19 |
| Patented | 4835 | Phase 2a | 12 |
| Phase 2 | 3490 | Registered | 12 |
| Phase 1 | 3372 | Discontinue in Phase 1 Trial | 8 |
| Approved | 3287 | Phase 0 | 7 |
| Terminated | 1601 | Phase 2b | 6 |
| Phase 3 | 1534 | Phase 1b | 6 |
| Discontinued in Phase 2 | 1016 | Approved (orphan drug) | 4 |
| Phase 1/2 | 976 | NDA filed | 4 |
| Discontinued in Phase 1 | 700 | Discontinued in Phase 2/3 | 3 |
| Preclinical | 554 | Discontinued in Phase 4 | 2 |
| Discontinued in Phase 3 | 234 | Phase 1/2a | 2 |
| Phase 2/3 | 172 | Phase 1b/2a | 2 |
| Clinical trial | 69 | Approval submitted | 1 |
| Withdrawn from market | 62 | BLA submitted | 1 |
| Phase 4 | 59 | Discontinue in Phase 2 Trial | 1 |
| Discontinued in Phase 1/2 | 54 | Discontinue in Phase 3 Trial | 1 |
| Discontinued in Preregistration | 42 | Discontinued in Phase 2a | 1 |
| Phase 2/3 Trial | 34 | Discontinued in Phase 2b | 1 |
| Phase 1/2 Trial | 30 | IND submitted | 1 |
| Application submitted | 29 |  |  |

**Table S2. Statistics and analysis result table for RNA-seq gene expression profiling data derived from The Cancer Genome Atlas (TCGA)**

| **Cancer Type** | **Cancer Name** | **Number of cancer samples** | **Number of normal samples** | **Number of up-regulated genes** | **Number of down-regulated genes** | **Whether it is included in DrugRepoBank** |
| --- | --- | --- | --- | --- | --- | --- |
| BLCA | Bladder Cancer | 407 | 19 | 124 | 487 | Yes |
| BRCA | Breast Cancer | 1104 | 114 | 107 | 495 |  |
| CESC | Cervical Cancer | 305 | 3 | 765 | 975 |  |
| CHOL | Bile Duct Cancer | 36 | 9 | 682 | 648 |  |
| COAD | Colon Cancer | 288 | 41 | 192 | 754 |  |
| COADREAD | Colon and Rectal Cancer | 383 | 51 | 192 | 746 |  |
| ESCA | Esophageal Cancer | 185 | 11 | 277 | 510 |  |
| HNSC | Head and Neck Cancer | 522 | 44 | 119 | 306 |  |
| KICH | Kidney Chromophobe | 66 | 25 | 176 | 1349 |  |
| KIRC | Kidney Clear Cell Carcinoma | 534 | 72 | 337 | 731 |  |
| KIRP | Kidney Papillary Cell Carcinoma | 291 | 32 | 175 | 873 |  |
| LIHC | Liver Cancer | 373 | 50 | 158 | 279 |  |
| LUAD | Lung Adenocarcinoma | 517 | 59 | 217 | 340 |  |
| LUNG | Lung Cancer | 1019 | 110 | 351 | 471 |  |
| LUSC | Lung Squamous Cell Carcinoma | 502 | 51 | 586 | 733 |  |
| PADD | Pancreatic Cancer | 179 | 4 | 169 | 42 |  |
| PCPG | Pheochromocytoma & Paraganglioma | 184 | 3 | 768 | 527 |  |
| PRAD | Prostate Cancer | 498 | 52 | 45 | 75 |  |
| READ | Rectal Cancer | 95 | 10 | 211 | 802 |  |
| SARC | Sarcoma | 263 | 2 | 134 | 469 |  |
| SKCM | Melanoma | 473 | 1 | 210 | 123 |  |
| STAD | Stomach Cancer | 415 | 35 | 114 | 260 |  |
| THCA | Thyroid Cancer | 513 | 59 | 185 | 275 |  |
| THYM | Thymoma | 120 | 2 | 143 | 326 |  |
| UCEC | Endometrioid Cancer | 177 | 24 | 280 | 554 |  |
| ACC | Adrenocortical Cancer | 79 | 0 | NA | NA | No |
| DLBC | Large B-cell Lymphoma | 48 | 0 | NA | NA |  |
| GBM | Glioblastoma | 172 | 0 | NA | NA |  |
| GBMLGG | lower grade glioma and glioblastoma | 702 | 0 | NA | NA |  |
| LAML | Acute Myeloid Leukemia | 173 | 0 | NA | NA |  |
| LGG | Lower Grade Glioma | 530 | 0 | NA | NA |  |
| MESO | Mesothelioma | 87 | 0 | NA | NA |  |
| OV | Ovarian Cancer | 308 | 0 | NA | NA |  |
| TGCT | Testicular Cancer | 156 | 0 | NA | NA |  |
| UCS | Uterine Carcinosarcoma | 57 | 0 | NA | NA |  |
| UVM | Ocular melanomas | 80 | 0 | NA | NA |  |

**Table S3.** Advantages and limitations of drug repositioning methodologies incorporated in DrugRepoBank.

| **Methods class** | **Specific method** | **Detail** | **Advantages** | **Limitations** | **Ref** |
| --- | --- | --- | --- | --- | --- |
| Similarity-based methods (Drug-drug similarity prediction) | Chemical structure similarity | Chemical structure similarity is estimated with atom pairs using the Tanimoto coefficient, which is defined as the proportion of atom pairs shared among two compounds divided by their union. | - Pharmacophore overlap. Directly reflects pharmacophore similarity, indicating potential overlap in binding sites and mechanisms of action. | - Structure-biology disconnect. May overlook drugs with distinct structures but similar biological effects due to alternative binding modes or allosteric regulation. | [[1](#_ENREF_1)] |
|  | Target protein sequence-based similarity | Pairwise protein sequence comparison is performed using the standard Needleman-Wunsch dynamic programming algorithm for global alignment, and the percentage of pairwise sequence identity is reported as the corresponding sequence similarity. | - Shared therapeutic potential. Identifies drugs with similar target profiles, suggesting shared therapeutic potential. | - Sequence-function disconnect. Sequence similarity alone may not fully capture functional or regulatory differences between targets. | [[2](#_ENREF_2)] |
|  | Target Protein functional similarity (GO Cellular Component (CC)) | Each drug was annotated with enriched GO Cellular Component (CC) terms and the functional similarity between any two drugs is determined by the semantic similarity of their associated GO terms using the topology of the GO graph structure. | - Functional commonality. Identifies shared functional attributes of drug targets across the three GO domains (Cellular Component, Molecular Function, and Biological Process), revealing potential similarities in their roles within cellular systems, molecular activities, and broader biological contexts. | - Annotation incompleteness. Functional annotations may be incomplete or outdated, limiting the accuracy of similarity calculations. | [[3](#_ENREF_3)] |
|  | Target Protein functional similarity (GO Molecular Function (MF)) | Each drug was annotated with enriched GO Molecular Function (MF) terms and the functional similarity between any two drugs is determined by the semantic similarity of their associated GO terms using the topology of the GO graph structure. |  |  |  |
|  | Target Protein functional similarity (GO Biological Process (BP)) | Each drug was annotated with enriched GO Biological Process (BP) terms and the functional similarity between any two drugs is determined by the semantic similarity of their associated GO terms using the topology of the GO graph structure. |  |  |  |
|  | Drug-induced pathway similarity | Pairwise similarity between any two pathways was estimated based on the similarity of their constituent genes using dice similarity. | - Systemic drug impact. Captures the systemic impact of drugs on cellular signaling and metabolic pathways, relevant for repositioning across diverse disease contexts. | - Annotation precision. Pathway annotations and drug-pathway associations may be incomplete or imprecise, affecting the accuracy of similarity calculations. | [[4](#_ENREF_4)] |
| Similarity-based method | Target-target similarity | Pairwise target protein sequences are compared based on the Needleman-Wunsch algorithm, which is designed based on dynamic programming. | - Homology-driven repositioning. Directly reflects the evolutionary relationship and functional conservation between drug targets, indicating potential for repositioning based on shared biological roles and mechanisms. | - Sequence-function disconnect. Sequence similarity may not always directly translate into functional equivalence or shared therapeutic potential, particularly when considering distant homologs or targets with divergent functions despite conserved sequences. | [[2](#_ENREF_2)] |
| Artificial intelligence-based methods | CPI_Prediciton | CPI_Prediciton is a CPI prediction approach by combining a graph neural network (GNN) for compounds and a convolutional neural network (CNN) for proteins. | - Attention-based interpretability. The use of a neural attention mechanism in CPI_Prediction enables the identification of important subsequences in a protein that are more relevant for a given drug compound, providing valuable insights into the molecular basis of drug repositioning candidates and aiding in the prioritization and rational design of repurposed drugs. | - Limited generalizability. While CPI_Prediction shows promising performance on diverse datasets, its ability to generalize to novel drug repositioning scenarios may be influenced by the diversity and representativeness of the training data, potentially limiting its applicability to certain target classes or chemical spaces. | [[5](#_ENREF_5)] |
|  | TransformerCPI | TransformerCPI is a sequence-based deep learning method with a self-attention mechanism for compound-protein interaction prediction. | - Attention-guided optimization. The model’s interpretable attention weights highlight crucial interacting regions in protein sequences and compound atoms, guiding medicinal chemists in refining drug structures for improved affinity and selectivity towards new targets, facilitating drug optimization during repositioning. - Cross-species applicability. TransformerCPI’s strong performance across human and non-human datasets suggests its potential for predicting drug repositioning opportunities across species, broadening the translational potential of repurposed drugs. | - Data availability constraints. Successful application of TransformerCPI for drug repositioning relies on the availability of comprehensive and accurately annotated compound and protein sequence data, which may not be uniformly accessible or complete for all potential repositioning targets. - Potential generalization issues. Despite demonstrating robust performance on diverse datasets, TransformerCPI’s generalization to novel drug repositioning scenarios may be challenged by unique molecular features or binding mechanisms not well represented in the training data. | [[6](#_ENREF_6)] |
|  | CapBM-DTI | CapBM-DTI is a drug-target interaction prediction method with capsule network and transfer learning. | - Hierarchical feature learning. CapBM-DTI’s utilization of a capsule network to extract internal hierarchical representations of protein targets and drug molecules enhances the discrimination of DTIs, potentially revealing subtle similarities between existing drugs and novel targets, facilitating drug repositioning discoveries. - Contextual protein sequence encoding. The method’s employment of BERT for contextual sequence feature extraction from target proteins allows for the capture of nuanced semantic relationships among amino acids, which could reveal hidden target similarities and expand the scope of drug repositioning opportunities. | - Data dependency. The effectiveness of CapBM-DTI in drug repositioning is heavily dependent on the availability and quality of input data, including protein sequences, compound graphs, and experimentally validated DTI datasets, which may vary in coverage and accuracy across different therapeutic areas and research settings. - Generalization challenges. While CapBM-DTI exhibits robust performance on various experimentally validated datasets, its ability to generalize to novel drug repositioning scenarios may be limited by the diversity and representativeness of the training data, potentially leading to missed opportunities or reduced predictive accuracy for certain target classes or chemical spaces. | [[7](#_ENREF_7)] |
| Signature-based methods | GSEAweight0 | GSEAweight0 is derived from the KS-like statistic with weighted KS enrichment statistic (ES): p = 0. | - Robust enrichment analysis. Provide a solid statistical basis for identifying shared biological pathways or mechanisms across drug pairs, aiding in understanding drug repositioning potential. - Flexible weighting schemes. Allow for customization based on biological assumptions, enhancing the relevance of findings for specific drug repositioning scenarios. | - Ambiguous weighting scheme selection. The choice of weighting strategy may not always be clear-cut, potentially impacting the accuracy and relevance of drug repositioning predictions. - Increased sensitivity to noise (GSEAweight2). Squaring fold changes can amplify the influence of noisy or irrelevant expression changes, potentially compromising the identification of true drug repositioning opportunities. - Limited focus on top-ranked genes. May overlook important but less pronounced expression changes that contribute to drug-disease relationships, reducing the method’s sensitivity in some repositioning scenarios. | [[8](#_ENREF_8)] |
|  | GSEAweight1 | GSEAweight1 is derived from the KS-like statistic with weighted KS enrichment statistic (ES): p = 1. |  |  |  |
|  | GSEAweight2 | GSEAweight2 is derived from the KS-like statistic with weighted KS enrichment statistic (ES): p = 2. |  |  |  |
|  | KS | KS is derived from the KS-like statistic with the rank of fold changes as weight. | - Non-parametric approach. Applicable to diverse L1000 datasets without assumptions about data distribution, increasing method versatility in drug repositioning contexts. - Global rank distribution comparison. Captures broad similarities in transcriptional profiles, potentially revealing overlooked repositioning opportunities. | - Insensitive to local expression changes. May miss subtle but critical shifts in gene expression patterns that underlie successful drug repositioning. - No gene-weighting. Treating all genes equally may underestimate the importance of strongly altered genes in driving drug repositioning potential. | [[9](#_ENREF_9)] |
|  | XSum | The XSum method was focused on the top genes ranked by fold changes of gene expression. | - Focus on top genes. Emphasizes the most significantly altered genes, which often drive therapeutic responses, improving the likelihood of identifying effective drug repositioning candidates. - Efficient summarization. Condenses complex expression data into a manageable format, facilitating decision-making in drug repositioning projects. | - Potential loss of information. By focusing exclusively on the top genes, XSum may neglect important but less pronounced changes that contribute to drug repositioning opportunities. - Sensitivity to fold change ranking and noise. Relying solely on fold change rankings can make the method susceptible to noise or experimental artifacts, potentially leading to inaccurate repositioning predictions. | [[10](#_ENREF_10)] |
|  | ZhangScore | The rank-based weights are set to all genes in one gene signature in ZhangScore. | - Rank-based weighting. Balances emphasis on the most impactful genes with consideration of overall expression patterns, potentially increasing the accuracy of drug repositioning predictions. - Superior performance. Demonstrated higher accuracy in identifying true positive drug pairs, suggesting increased reliability in pinpointing promising repositioning candidates. | - Reliance on fold change ranking. Sensitivity to noise or experimental biases in gene expression measurements may affect the accuracy of drug repositioning similarity scores. - More complex calculation. Compared to simpler methods like KS or GSEAweight0, ZhangScore’s algorithm may require more computational resources, potentially impacting the efficiency of large-scale drug repositioning screens. | [[11](#_ENREF_11)] |
| Network-based method | DRviaSPCN | DRviaSPCN is an approach to prioritize cancer candidate drugs by considering drug-induced subpathways and their crosstalk effects. | - Subpathway crosstalk insights. Directly explores the interplay between subpathways (SPs) in cancer, providing a unique angle for drug repositioning by considering SP cooperation in cell survival and drug resistance. - Quantitative SP analysis. Establishes an SP network with centrality scores to quantify SP influence and calculates drug-disease associations at the SP level, incorporating SP functionality and crosstalk effects. | - Methodological complexity. Involves multiple intricate steps (SP extraction, network construction, centrality scoring, enrichment analysis), which may increase computational demands and necessitate specialized knowledge. - Data quality dependence. The performance of DRviaSPCN is tied to the availability and accuracy of input data, such as KEGG pathways, GO terms, and gene expression profiles. | [[12](#_ENREF_12)] |
|  | DrugSim2DR | DrugSim2DR is a tool that systematically predicts drug functional similarities within the context of specific diseases to facilitate drug repurposing. | - Disease-specific drug functional similarity. Directly assesses drug-drug functional similarity within the context of a specific disease state, capturing disease-relevant molecular characteristics ignored by general similarity measures, enabling more accurate prediction of drug-drug relationships and potential drug combinations. - Transcriptional dysregulation consideration. Incorporates disease-induced transcriptional dysregulation of target genes into the functional similarity assessment, refining the understanding of drug actions in the disease context. | - Data availability constraints. The performance and applicability of DrugSim2DR depend on the availability and quality of disease-specific transcriptional data, drug target information, and functional annotations, which may vary across diseases and research settings. - Computational complexity. The approach involves constructing and analyzing complex networks, calculating functional similarities, and performing network propagation algorithms, which can be computationally intensive and require specialized computational resources. |  |

Table S4. MOA categories (Recurring twice or more) and corresponding drugs in Top50 candidates as predicted by six Signature-based methods and two network-based methods

| **Cancer type** | **Signature-based methods** | | | **DRviaSPCN** | | | **DrugSim2DR** | | |
| --- | --- | --- | --- | --- | --- | --- | --- | --- | --- |
|  | **Top MOA** | **Drugs** | **Number** | **Top MOA** | **Drugs** | **Number** | **Top MOA** | **Drugs** | **Number** |
| BLCA | HDAC inhibitor | Vorinostat, Scriptaid | 8 | Antibiotics | Rifabutin, Cefoperazone | 2 | Anticholinergic agents | Methantheline, Oxyphencyclimine, Anisotropine Methylbromide, Dicyclomine | 4 |
|  | MEK inhibitor | Selumetinib | 4 | HDAC inhibitor | Scriptaid, Vorinostat | 2 | Antispasmodics | Tolterodine, Solifenacin, Scopolamine | 3 |
|  |  |  |  | Microtubule inhibitor / Tubulin polymerization inhibitor | Vinblastine, Podophyllotoxin | 2 | Antiparkinson agents | Metixene, Trihexyphenidyl | 2 |
|  |  |  |  |  |  |  | Antipsychotic agents | Clozapine, Quetiapine | 2 |
|  |  |  |  |  |  |  | Acetylcholine receptor antagonist | Darifenacin, Hyoscyamine | 2 |
|  |  |  |  |  |  |  | Antiarrhythmic agents | Atropine, Disopyramide | 2 |
| BRCA | CDK inhibitor | Palbociclib | 2 | Antibiotics | Kanamycin, Netilmicin | 2 | Vasoconstrictor agents | Sumatriptan, Naratriptan | 2 |
|  |  |  |  | Antipsychotic agents | Perphenazine, Loxapine | 2 |  |  |  |
|  |  |  |  | Anticancer agents | Lomustine, Streptozocin | 2 |  |  |  |
|  |  |  |  | Glucocorticoid receptor agonist | Fludroxycortide, Halcinonide | 2 |  |  |  |
| CESC | HDAC inhibitor | Vorinostat | 23 | Antipsychotic agents | Clozapine, Perphenazine, Trifluoperazine, Fluphenazine, Thioridazine, Prochlorperazine, Chlorpromazine | 7 | Antihypertensive agents | Phenoxybenzamine, Bethanidine, Labetalol, Bevantolol, Nebivolol | 5 |
|  | MEK inhibitor | Selumetinib | 2 | Antibiotics | Rifabutin, Oxytetracycline | 2 | Adrenergic receptor antagonist | Carvedilol, Propranolol, Pindolol, Oxprenolol | 4 |
|  |  |  |  | Antidepressants | Trazodone, Doxepin | 2 | Adrenergic receptor agonist | Epinephrine, Norepinephrine, Terbutaline | 3 |
|  |  |  |  |  |  |  | Bronchodilator agents | Salmeterol, Salbutamol, Fenoterol | 3 |
|  |  |  |  |  |  |  | Antiinflammatory agents | Fluticasone, Dexamethasone | 2 |
|  |  |  |  |  |  |  | Cardiotonic agents | Dobutamine, Arbutamine | 2 |
| CHOL | HDAC inhibitor | Vorinostat, Panobinostat | 4 | Antihypertensive agents | Phenoxybenzamine, Lisinopril | 2 |  |  |  |
|  | Protein synthesis inhibitor | Puromycin | 3 | Anticancer agents | Lomustine, Letrozole | 2 |  |  |  |
|  | Topoisomerase inhibitor | Etoposide, Irinotecan | 3 | Antidepressants | Trazodone, Tranylcypromine | 2 |  |  |  |
|  | CDK inhibitor | Palbociclib, Alvocidib | 2 | HDAC inhibitor | Vorinostat, Scriptaid | 2 |  |  |  |
| COAD |  |  |  | Antihypertensive agents | Betaxolol, Bisoprolol, Carteolol, Labetalol | 4 | Antihypertensive agents | Isradipine, Nitrendipine, Captopril | 3 |
|  |  |  |  | Antibiotics | Rifabutin, Amoxicillin | 2 | Analgesics | Magnesium Sulfate, Nifedipine | 2 |
|  |  |  |  | Glucocorticoid receptor agonist | Methylprednisolone, Rimexolone | 2 |  |  |  |
|  |  |  |  | Antiinflammatory agents | Valdecoxib, Fluticasone | 2 |  |  |  |
| COADREAD | Estrogen receptor antagonist | Fulvestrant | 2 | Antiinflammatory agents | Rofecoxib, Valdecoxib, Fluticasone | 3 | Antihypertensive agents | Isradipine, Nitrendipine, Captopril | 3 |
|  |  |  |  | Antihypertensive agents | Betaxolol, Carteolol | 2 | Analgesics | Magnesium Sulfate, Nifedipine | 2 |
| ESCA | HDAC inhibitor | Vorinostat | 3 | Antihypertensive agents | Indapamide, Benzthiazide, Iloprost, Metolazone | 4 | Anticancer agents | Cabergoline, Mitotane | 2 |
|  | MEK inhibitor | Selumetinib | 2 | Antibiotics | Ampicillin, Rifabutin, Nafcillin | 3 | Antiparkinson agents | Bromocriptine, Lisuride | 2 |
|  |  |  |  | Adenosine receptor antagonist | Theophylline, Sulmazole | 2 | Sodium channel blocker | Oxcarbazepine, Brivaracetam | 2 |
| HNSC | HDAC inhibitor | Vorinostat, Entinostat | 2 | Antiinflammatory agents | Fluticasone, Clobetasol | 2 | Antimigraine agents | Dihydroergotamine, Eletriptan | 2 |
|  |  |  |  | Antihypertensive agents | Betaxolol, Isradipine | 2 | Anticonvulsants | Topiramate, Acetazolamide | 2 |
|  |  |  |  | Anticancer agents | Streptozocin, Letrozole | 2 | Vasoconstrictor agents | Sumatriptan, Naratriptan | 2 |
|  |  |  |  |  |  |  | KIT inhibitor / PDGFR tyrosine kinase receptor inhibitor / VEGFR inhibitor | Pazopanib, Vatalanib | 2 |
| KICH | HDAC inhibitor | Vorinostat, Entinostat | 6 | Topoisomerase inhibitort | Irinotecan, Camptothecin, Daunorubicin | 3 | Anticonvulsants | Metharbital, Primidone, Lamotrigine, Acetazolamide | 4 |
|  |  |  |  | Antibiotics | Sulfanilamide, Amikacin | 2 | Antihypertensive agents | Benzthiazide, Cyclothiazide, Dorzolamide | 3 |
|  |  |  |  | Antihypertensive agents | Phenoxybenzamine, Labetalol | 2 | Analgesics | Etomidate, Butalbital | 2 |
|  |  |  |  | Estrogen receptor antagonist | Tamoxifen, Clomifene | 2 | Anesthetics | Isoflurane, Desflurane | 2 |
|  |  |  |  |  |  |  | Keratolytic agents | Acitretin, Tretinoin | 2 |
|  |  |  |  |  |  |  | Diuretics | Hydroflumethiazide, Ethoxzolamide | 2 |
|  |  |  |  |  |  |  | Anticancer agents | Alitretinoin, Diethylstilbestrol | 2 |
|  |  |  |  |  |  |  | Retinoid receptor agonist | Bexarotene, Adapalene | 2 |
| KIRC | Topoisomerase inhibitor | Amsacrine, Teniposide, Irinotecan, Etoposide, Doxorubicin | 7 | Antibiotics | Oxytetracycline, Colistin, Rolitetracycline, Nafcillin, Cefoperazone | 5 | Analgesics | Codeine, Hydromorphone, Oxycodone, Butorphanol, Nalbuphine, Levorphanol, Alvimopan, Buprenorphine, Morphine, Dextropropoxyphene | 10 |
|  | Estrogen receptor antagonist | Fulvestrant, Tamoxifen | 3 | Adenosine receptor antagonist | Sulmazole, Theophylline | 2 | Anesthetics | Sufentanil, Pentazocine | 2 |
|  | CDK inhibitor | Palbociclib | 2 |  |  |  |  |  |  |
|  | MEK inhibitor | Selumetinib | 2 |  |  |  |  |  |  |
| KIRP | HDAC inhibitor | Scriptaid, Vorinostat | 4 | Antimalarials | Halofantrine, Proguanil | 2 |  |  |  |
|  | Topoisomerase inhibitor | Teniposide, Amsacrine, Daunorubicin, Mitoxantrone | 4 |  |  |  |  |  |  |
|  | CDK inhibitor | Palbociclib, Alvocidib | 2 |  |  |  |  |  |  |
| LIHC | Topoisomerase inhibitor | Amsacrine, Teniposide, Irinotecan, Etoposide | 6 | Antihypertensive agents | Lisinopril, Phenoxybenzamine | 2 | Antiallergic agents | Promethazine, Brompheniramine, Cinnarizine, Azelastine | 4 |
|  | HDAC inhibitor | Scriptaid, Vorinostat | 4 | Antidepressants | Paroxetine, Clomipramine | 2 | Antipsychotic agents | Loxapine, Clozapine, Quetiapine | 3 |
|  | MEK inhibitor | Selumetinib | 3 | Antipsychotic agents | Triflupromazine, Perphenazine | 2 | Antidepressants | Doxepin, Maprotiline | 2 |
|  | CDK inhibitor | Palbociclib | 2 | Histamine receptor antagonist | Terfenadine, Astemizole | 2 | Neuromuscular nondepolarizing agents | Tubocurarine, Rocuronium | 2 |
|  | Protein synthesis inhibitor | Puromycin | 2 | Protein synthesis inhibitor | Mercaptopurine, Emetine | 2 | Analgesics | Amitriptyline, Succinylcholine | 2 |
|  |  |  |  |  |  |  | Histamine receptor antagonist | Terfenadine, Pheniramine | 2 |
| LUAD | HDAC inhibitor | Vorinostat | 4 | Anticancer agents | Mercaptopurine, Vinblastine, Streptozocin | 3 | Antipsychotic agents | Risperidone, Fluphenazine, Perphenazine, Thiothixene | 4 |
|  | Protein synthesis inhibitor | Thiostrepton, Puromycin | 3 | Antihypertensive agents | Mecamylamine, Rescinnamine, Nadolol | 3 | Vasoconstrictor agents | Methysergide, Sumatriptan, Naratriptan | 3 |
|  | MEK inhibitor | Selumetinib | 2 | Hypoglycemic agents | Pioglitazone, Chlorpropamide | 2 | Antimigraine agents | Dihydroergotamine, Eletriptan | 2 |
|  | RAF inhibitor | Dabrafenib, Vemurafenib | 2 |  |  |  |  |  |  |
| LUNG | HDAC inhibitor | Vorinostat | 9 | Antihypertensive agents | Enalapril, Mecamylamine, Nadolol | 3 |  |  |  |
|  | Topoisomerase inhibitor | Amsacrine, Doxorubicin, Etoposide | 4 |  |  |  |  |  |  |
|  | MEK inhibitor | Selumetinib | 2 |  |  |  |  |  |  |
|  | CDK inhibitor | Palbociclib | 2 |  |  |  |  |  |  |
|  | Protein synthesis inhibitor | Puromycin, Thiostrepton | 2 |  |  |  |  |  |  |
| LUSC | HDAC inhibitor | Vorinostat, Entinostat | 15 | Microtubule inhibitor / Tubulin polymerization inhibitor | Vinblastine, Podophyllotoxin | 2 |  |  |  |
|  | MEK inhibitor | Selumetinib | 2 | Antidepressants | Citalopram, Trimipramine | 2 |  |  |  |
|  | Topoisomerase inhibitor | Amsacrine, Etoposide | 2 | Antifungal agents | Butoconazole, Ciclopirox | 2 |  |  |  |
| PADD | HDAC inhibitor | Vorinostat, Panobinostat | 19 | Histamine receptor antagonist | Terfenadine, Astemizole, Chloropyramine | 3 |  |  |  |
|  |  |  |  | Antiallergic agents | Triprolidine, Diphenhydramine | 2 |  |  |  |
|  |  |  |  | Antipsychotic agents | Perphenazine, Chlorprothixene | 2 |  |  |  |
| PCPG | RAF inhibitor | Dabrafenib, Vemurafenib | 2 | Antibiotics | Rifabutin, Nalidixic Acid, Cefmetazole, Colistin | 4 |  |  |  |
|  |  |  |  | HDAC inhibitor | Vorinostat, Scriptaid | 2 |  |  |  |
|  |  |  |  | Antiinflammatory agents | Suprofen, Fluticasone | 2 |  |  |  |
| PRAD | Topoisomerase inhibitor | Etoposide, Teniposide | 3 | Antidepressants | Doxepin, Maprotiline | 2 | HDAC inhibitor | Belinostat, Panobinostat, Vorinostat, Romidepsin | 4 |
|  |  |  |  | Antihypertensive agents | Iloprost, Acebutolol | 2 | Antidepressants | Mianserin, Milnacipran | 2 |
|  |  |  |  | Antipsychotic agents | Loxapine, Perphenazine | 2 |  |  |  |
|  |  |  |  | Microtubule inhibitor / tubulin polymerization inhibitor | Vinblastine, Podophyllotoxin | 2 |  |  |  |
| READ |  |  |  | Antibiotics | Kanamycin, Rifabutin, Sulfanilamide | 3 | Anticholinergic agents | Oxyphencyclimine, Anisotropine Methylbromide, Dicyclomine | 3 |
|  |  |  |  |  |  |  | Antipsychotic agents | Triflupromazine, Molindone | 2 |
| SARC | Topoisomerase inhibitor | Amsacrine, Doxorubicin, Daunorubicin, Teniposide | 5 | Antibiotics | Rifabutin, Gentamicin, Cefadroxil, Oxytetracycline | 4 | Antipsychotic agents | Risperidone, Zuclopenthixol, Haloperidol, Carphenazine, Thioridazine, Thiothixene, Perphenazine, Fluphenazine | 8 |
|  |  |  |  | HDAC inhibitor | Vorinostat, Scriptaid | 2 | Analgesics | Ergotamine, Magnesium Sulfate | 2 |
|  |  |  |  | Topoisomerase inhibitor | Mitoxantrone, Daunorubicin | 2 | Hypoglycemic agents | Acarbose, Miglitol | 2 |
| SKCM |  |  |  | Antipsychotic agents | Fluspirilene, Promazine, Chlorprothixene | 3 | Antihypertensive agents | Clonidine, Phenoxybenzamine, Fenoldopam, Phentolamine, Labetalol | 5 |
|  |  |  |  | Antiviral agents | Ifenprodil, Amantadine | 2 | Acetylcholine receptor agonist | Norepinephrine, Epinephrine, Oxymetazoline | 3 |
|  |  |  |  | Hypoglycemic agents | Gliclazide, Acetohexamide | 2 | Adrenergic receptor antagonist | Prazosin, Carvedilol, Dronedarone | 3 |
| STAD |  |  |  | Antibiotics | Rifabutin, Troleandomycin, Chloramphenicol | 3 | Antihypertensive agents | Phenoxybenzamine, Fenoldopam, Bethanidine | 3 |
|  |  |  |  | Antiallergic agents | Cetirizine, Ketotifen | 2 | Antiparkinson agents | Pergolide, Bromocriptine | 2 |
|  |  |  |  | Adrenergic receptor antagonist | Propranolol, Dihydroergocristine | 2 |  |  |  |
| THCA |  |  |  | Antibiotics | Tobramycin, Chloramphenicol, Cefazolin | 3 | Antidepressants | Escitalopram, Maprotiline | 2 |
|  |  |  |  | Antiarrhythmic agents | Timolol, Sotalol | 2 | Antihypertensive agents | Clonidine, Treprostinil | 2 |
|  |  |  |  | Antiinflammatory agents | Valdecoxib, Flurbiprofen | 2 | HDAC inhibitor | Belinostat, Panobinostat | 2 |
| THYM |  |  |  | Antiallergic agents | Cetirizine, Ketotifen, Levocabastine | 3 | Anticancer agents | Lucanthone, Pemetrexed | 2 |
|  |  |  |  | Antibiotics | Minocycline, Amikacin | 2 | Antibiotics | Ampicillin, Ceftriaxone | 2 |
|  |  |  |  |  |  |  | Dietary supplement | Glycine, Pyruvic Acid | 2 |
|  |  |  |  |  |  |  | Ribonucleotide reductase inhibitor | Gemcitabine, Fludarabine | 2 |
| UCEC | HDAC inhibitor | Vorinostat | 8 |  |  |  | Antiparkinson agents | Ropinirole, Pergolide, Bromocriptine, Pramipexole | 4 |
|  | MEK inhibitor | Selumetinib | 3 |  |  |  | Antipsychotic agents | Prochlorperazine, Chlorpromazine, Haloperidol | 3 |
|  | Topoisomerase inhibitor | Amsacrine, Teniposide | 2 |  |  |  | Antihypertensive agents | Clonidine, Fenoldopam | 2 |
|  |  |  |  |  |  |  | Adrenergic receptor agonist | Xylazine, Oxymetazoline | 2 |

**Figure S1**. Workflow of the similarity-based drug repositioning. (A) Drug-drug similarity calculation process. (B) Target-target similarity calculation process


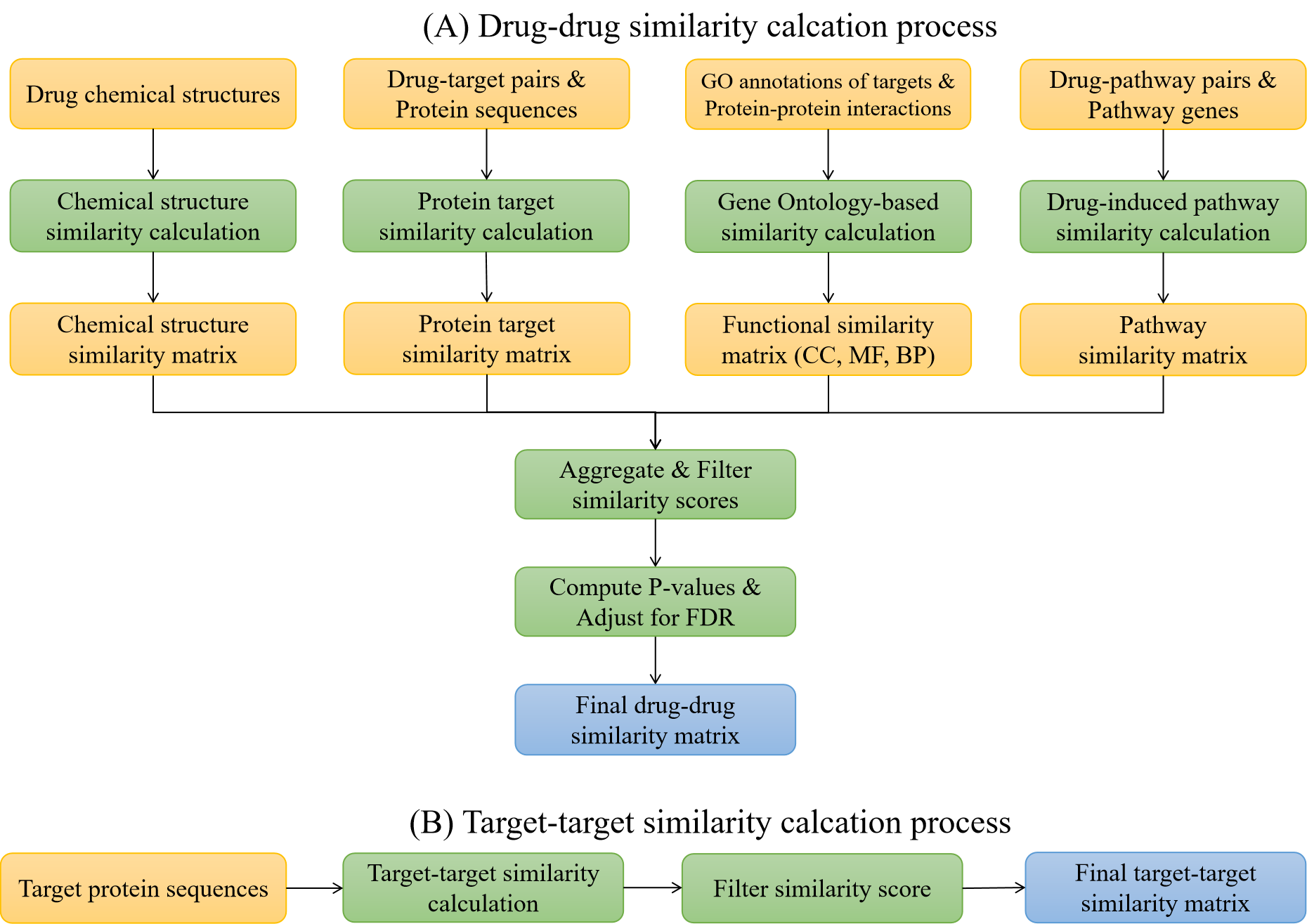


**Figure S2.** Workflow of the artificial intelligence-based drug repositioning.
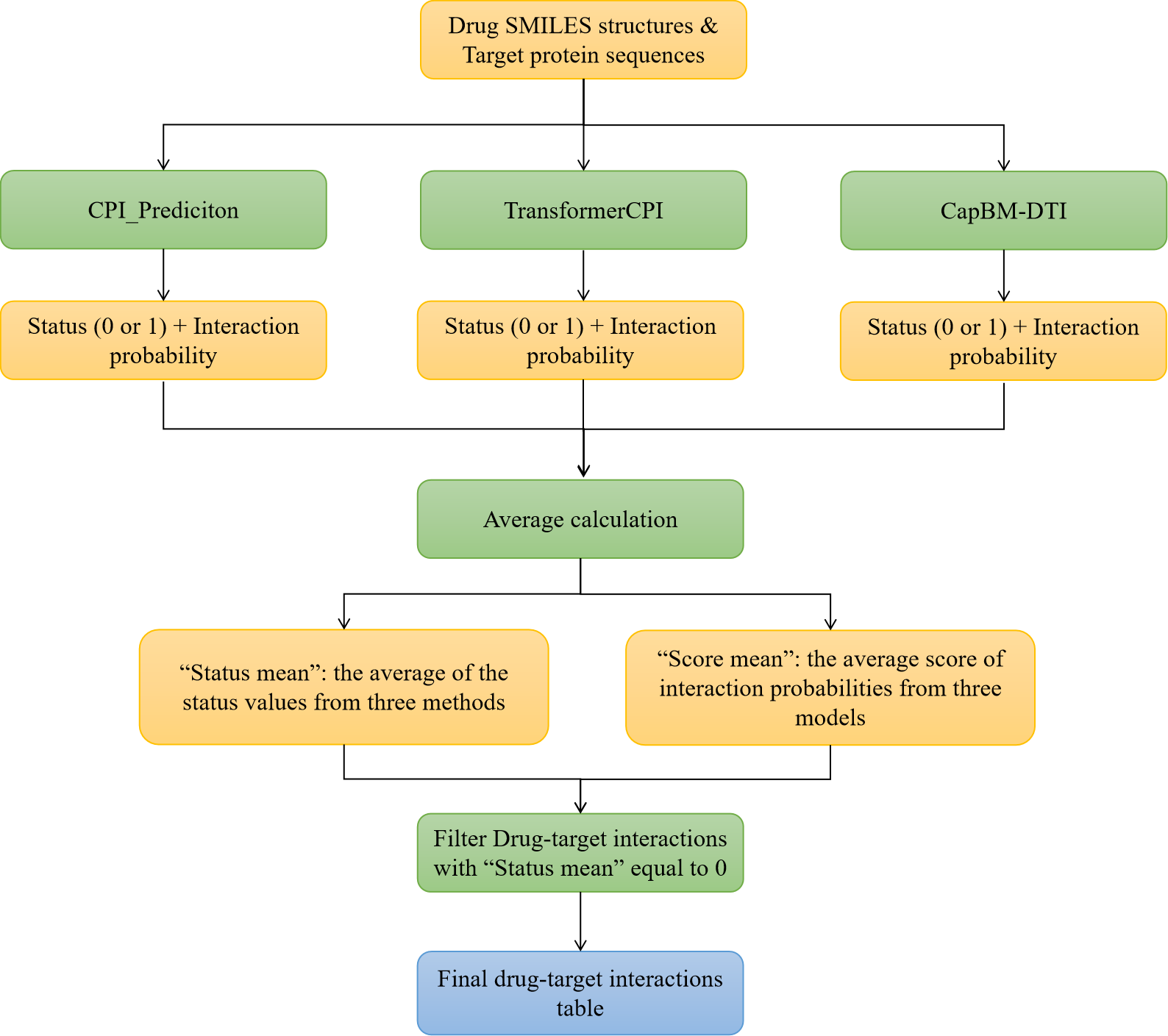


**Figure S3.** Workflow of the signature-based drug repositioning.


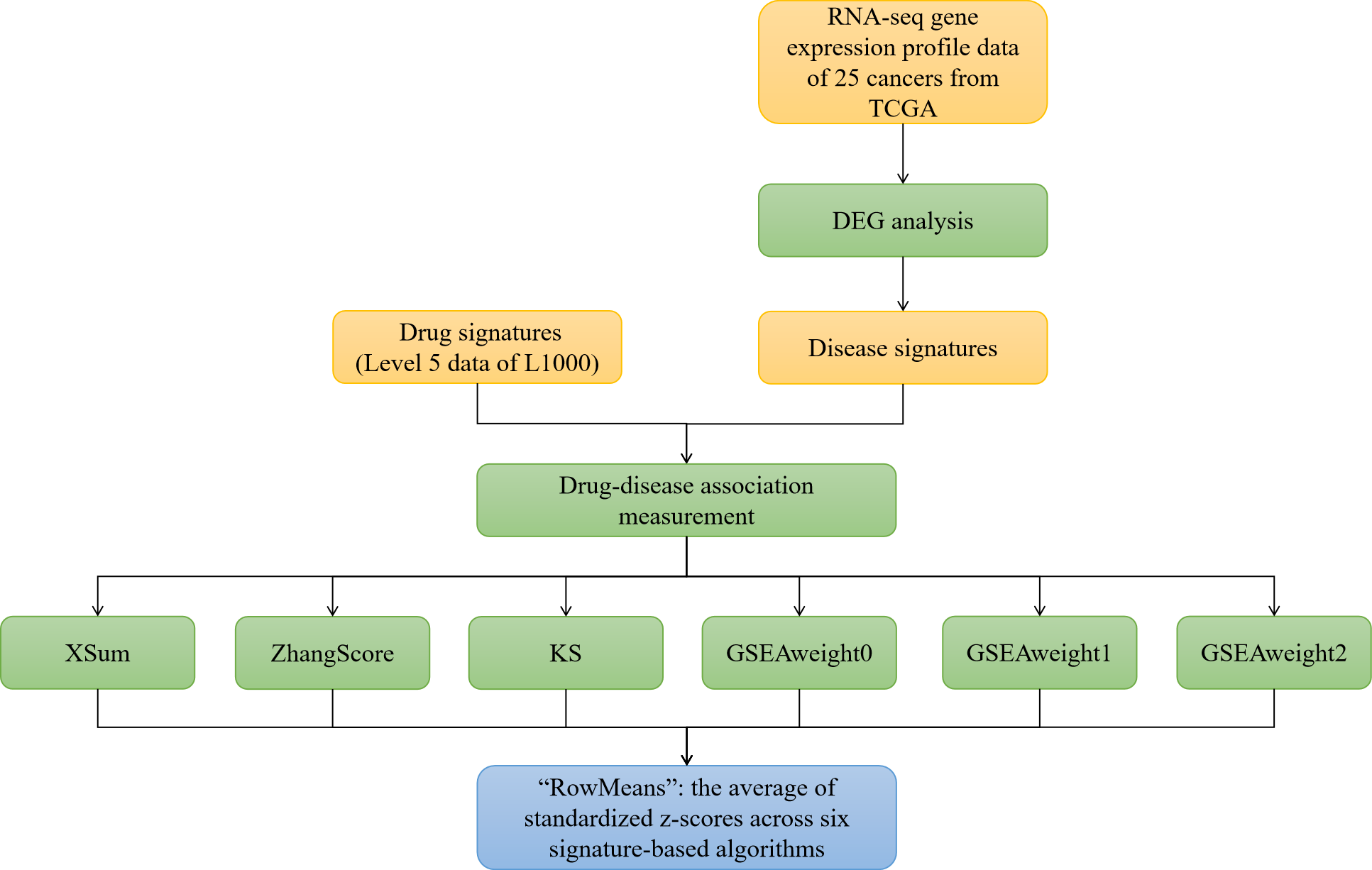


**Figure S4.** Workflow of the network-based drug repositioning.


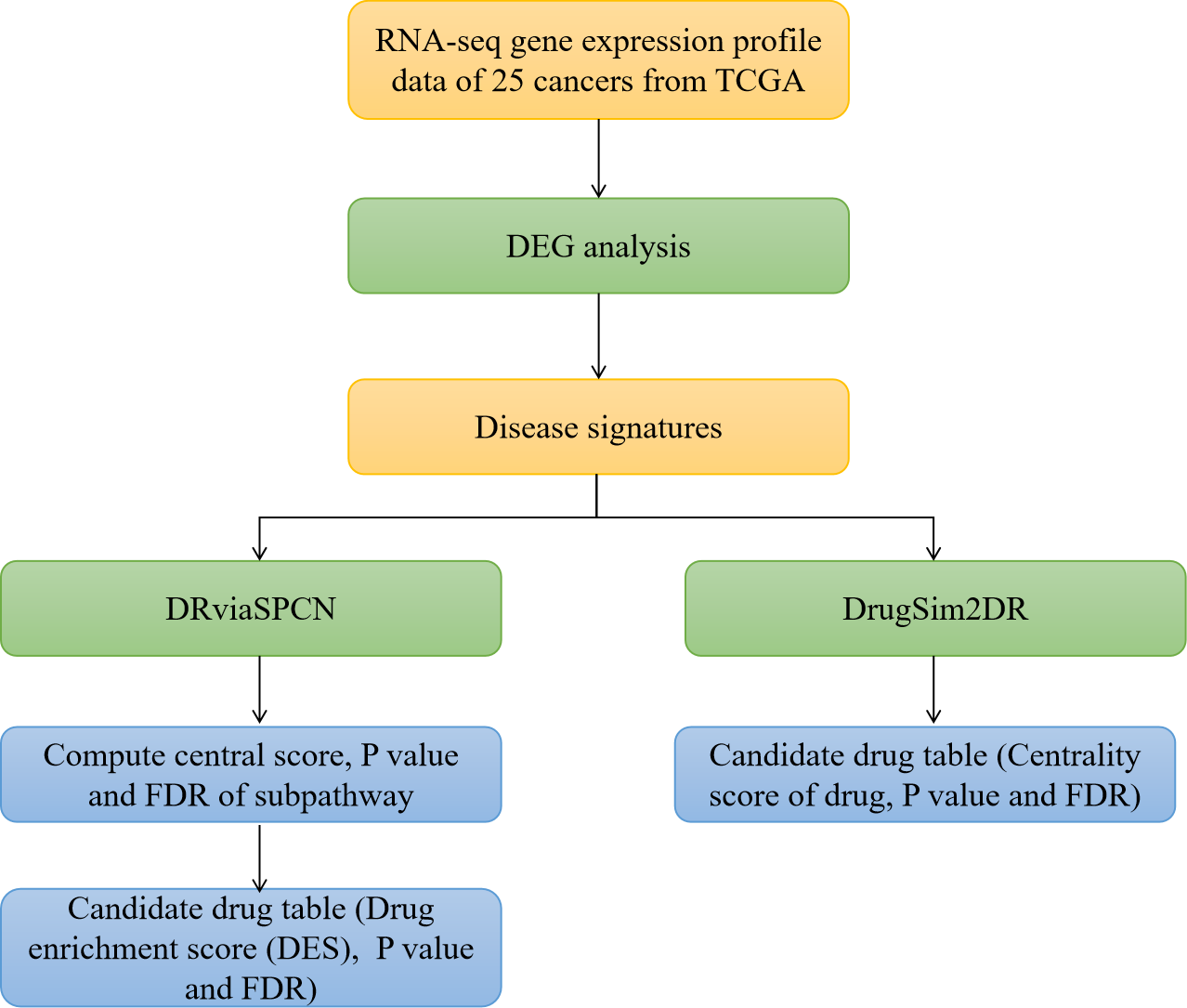


**Figure S5.** Venn diagram illustrating drug-target interactions predicted by three AI-based drug repositioning methods


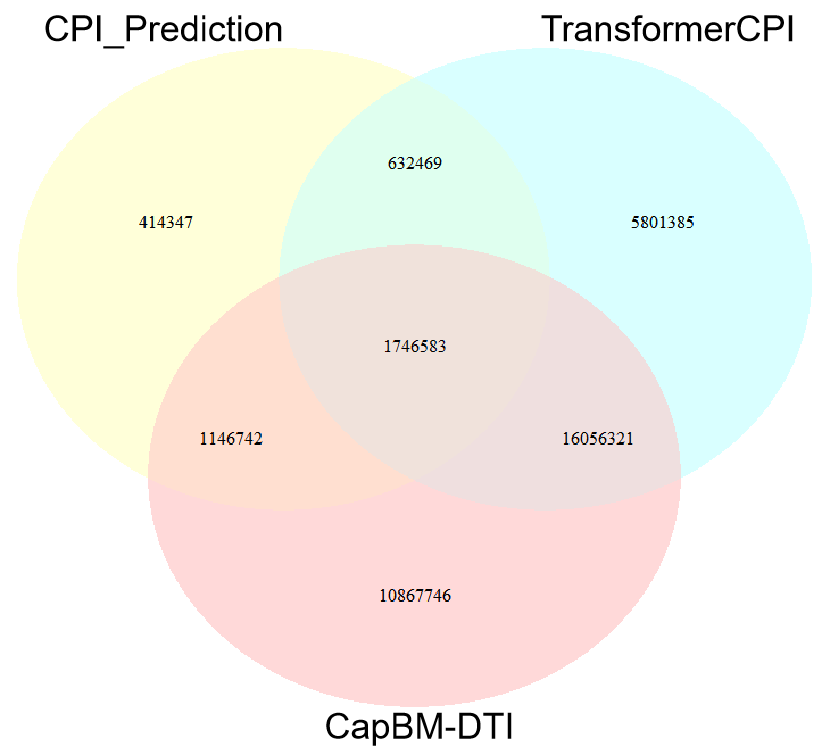


**Figure S6.** Upset plot depicting drug-drug similarity predictions from six signature-based drug repositioning approaches

**Figure S7.** An overview of the algorithm types used by the three search engines in DrugRepoBank’s Prediction Module. (A) Drug Search, (B) Target Search, and (C) Disease Search.


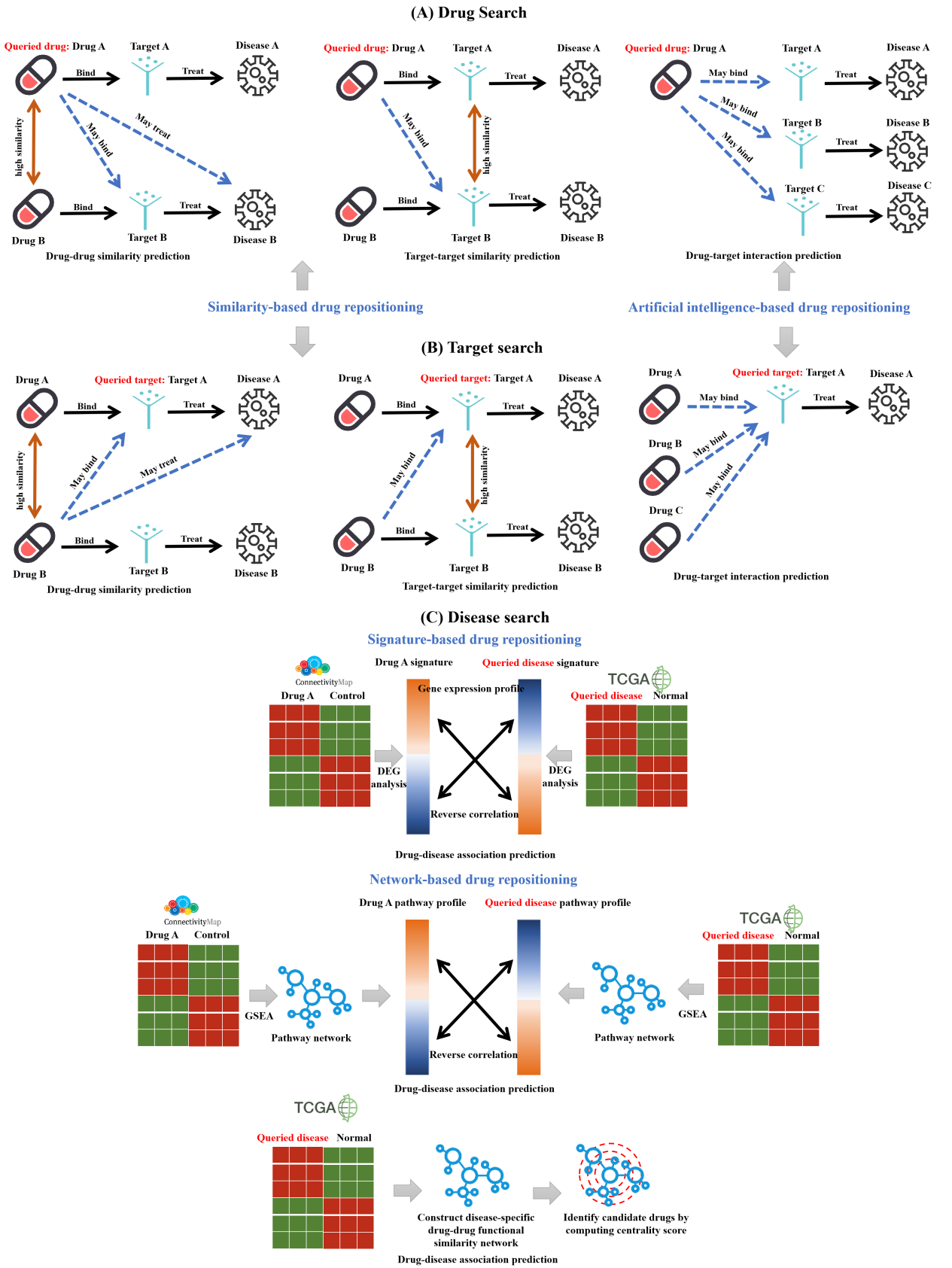


**Figure S8.** Detailed information on the web interface. There are two main modules: the “Prediction” module and the “Literature” module. The “Prediction” module consists of (A) Drug Search, (B) Target Search, and (C) Disease Search. The “Literature” module has a (D) literature search engine.


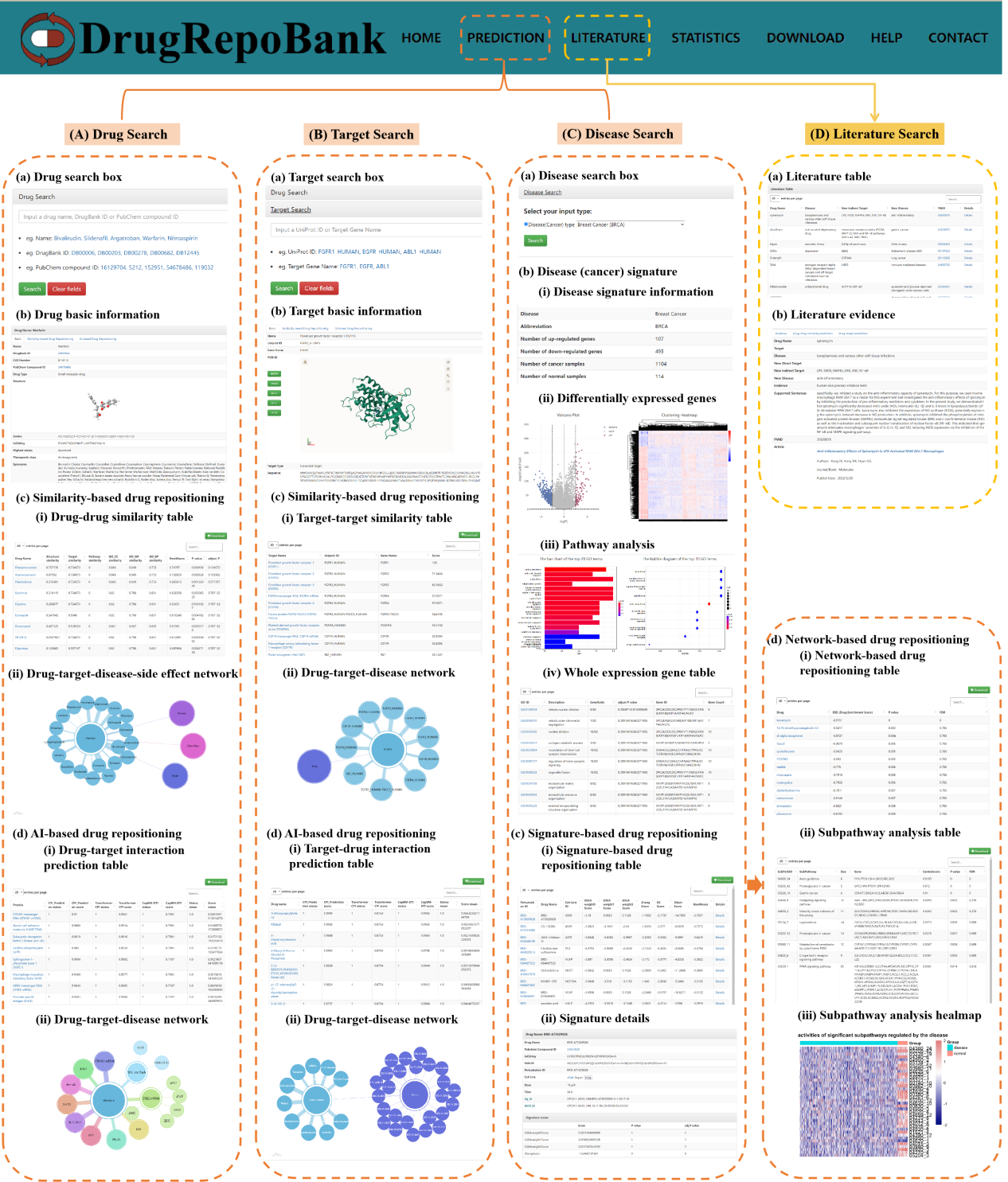


**1 Similarity-based drug repositioning algorithms**

The method of drug-drug similarity prediction encompasses chemical structure similarity [[1](#_ENREF_1)], target protein sequence-based similarity [[2](#_ENREF_2)], target protein functional similarity (GO Cellular Component (CC)) [[3](#_ENREF_3)], target protein functional similarity (GO Molecular Function (MF)) [[3](#_ENREF_3)], target protein functional similarity (GO Biological Process (BP)) [[3](#_ENREF_3)], and Drug-induced pathway similarity [[4](#_ENREF_4)]. The prediction of target-target similarity hinges on comparing target protein sequences, a process facilitated by utilizing the Needleman-Wunsch algorithm, which has been specifically crafted using dynamic programming techniques [[2](#_ENREF_2)].

**1.1 Drug-drug similarity prediction**

**1.1.1 Chemical structure similarity**

The Tanimoto coefficient estimates Drug-drug chemical structure similarity, as implemented by the ChemmineR [[1](#_ENREF_1)] package (version 3.54.0) within the R software.

$$S_{c}(d_{i},d_{j})=\frac{\left| {AP}_{i}\cap{AP}_{j} \right|}{\left| {AP}_{i}\cup{AP}_{j} \right|}$$

*AP_i_* represents atom pairs of drug *d_i_*_,_ and *AP_j_* means atom pairs of drug *d_j_*. The Tanimoto coefficient is the proportion of shared atom pairs in all-atom pairs of two compounds.

**1.1.2 Target protein sequence-based similarity**

Pairwise target protein sequences are compared based on the Needleman-Wunsch dynamic programming algorithm, which is calculated by the *Biostrings* [[2](#_ENREF_2)] package (version 2.70.3) in R software. One drug may target multiple target proteins, so this approach needs to consider the “best-match average”.

$$S_{t}(d_{i},d_{j})=\frac{\sum_{a\in T_{i}} \max_{\forall b\in T_{j}} \left\{ S\left( a,b \right) \right\}+\sum_{b\in T_{j}} \max_{\forall a\in T_{i}} \left\{ S\left( b,a \right) \right\}}{\left| T_{i} \right|*\left| T_{j} \right|}$$

*T_i_* and *T_j_* represent the target proteins of drug *d_i_* and *d_j_*, respectively. S(a, b) represents a symmetric sequence-based similarity measure between two protein targets, where *a* belongs to the set *T_i_*, and *b* belongs to the set *T_j_*. Each target in *T_i_* only matches with the most similar one of *d_j_* and vice versa.

**1.1.3 Target protein functional similarity**

Each drug is annotated with enriched GO Cellular Component (CC), Molecular Function (MF), and Biological Process (BP) terms. The target protein functional similarity between drug pairs is estimated based on the semantic similarity of associated GO terms of each drug, which is calculated by *the GOSemSim* package (version 2.28.1) in R [[3](#_ENREF_3)]. Since one drug may be annotated with multiple GO terms, the final semantic similarity should be calculated according to the “best-match average” [[5](#_ENREF_5)].

$$S_{f}(d_{i},d_{j})=\frac{\sum_{m\in T_{i}} \max_{\forall n\in T_{j}} \left\{ S\left( m,n \right) \right\}+\sum_{n\in T_{j}} \max_{\forall m\in T_{i}} \left\{ S\left( n,m \right) \right\}}{\left| T_{i} \right|*\left| T_{j} \right|}$$

*T_i_* and *T_j_* represent the target proteins of drug *d_i_* and *d_j_*, respectively. S(m, n) represents the semantic similarity of each target protein associated with GO terms, where *m* belongs to the set *T_i_*, and *n* belongs to the set *T_j_*. Each target in *T_i_* only matches with the most similar one of *d_j_* and vice versa.

**1.1.4 Drug-induced pathway similarity**

Pairwise pathway similarity is evaluated based on the similarity of constituent genes by using the Dice Similarity Coefficient (DSC) ranging from 0 to 1, estimated by the *BioCor* package (version 1.26.0) in R software [[4](#_ENREF_4)].

$$S_{p}(d_{i},d_{j})=\max_{\forall a\in P_{i},\forall b\in P_{j}} \left\{ \frac{2\left| a\cap b \right|}{\left| a \right|+\left| b \right|} \right\}$$

*P_i_* represents the pathways induced by drug *d_i_* and means the set of its constituent genes. Likewise, *P_j_* represents the pathways induced by the drug *d_j,_* and *b* means its constituent genes.

**1.2 Target-target similarity prediction**

Pairwise target protein sequences are compared based on the Needleman-Wunsch algorithm, designed based on dynamic programming. DrugRepoBank estimates the target-target similarity by the *Biostrings* [[2](#_ENREF_2)] R package (version 2.70.3). Values with scores equal to “NA” or less than 20 will be excluded.

**2 Artificial intelligence-based drug repositioning algorithms**

Incorporating three cutting-edge artificial intelligence-based drug repositioning algorithms, namely CPI_Prediction (https://github.com/masashitsubaki/CPI_prediction) [[6](#_ENREF_6)], TransformerCPI (https://github.com/lifanchen-simm/transformerCPI) [[7](#_ENREF_7)], and CapBM-DTI (https://github.com/huangyixian666/CapBM-DTI) [[10](#_ENREF_10)], into DrugRepoBank, we aim to fortify our platform’s capabilities for predicting compound-protein interactions and drug-target interactions (DTIs).

**2.1 CPI_Prediciton**

CPI_Prediciton [[6](#_ENREF_6)] is an innovative approach centered around end-to-end representation learning, applying deep neural networks to discrete symbolic data. Specifically, compounds are represented as graphs, with atoms as vertices and chemical bonds as edges, while proteins are treated as sequences of amino acids. The model combines a graph neural network (GNN) for compounds and a convolutional neural network (CNN) for proteins. Experiments on three CPI datasets reveal that CPI_Prediciton consistently achieves competitive or superior performance compared to existing CPI prediction methods, even excelling on unbalanced datasets.

**2.2 TransformerCPI**

TransformerCPI [[7](#_ENREF_7)] introduces a neural network model for predicting compound-protein interactions, building upon the Transformer architecture [[8](#_ENREF_8)]. This framework treats compounds and proteins as distinct sequence types to forecast interactions between them. To represent protein sequences in TransformerCPI, a sequence of 3-gram amino acid segments is generated by splitting the sequences, and these segments are then transformed into embeddings using the word2vec technique [[9](#_ENREF_9)]. These sequential feature vectors are subsequently processed through an encoder composed of a gated convolutional network incorporating Conv1D and gated linear units. The output from the encoder is then fed into a decoder. For compounds, TransformerCPI converts Simplified Molecular Input Line Entry System (SMILES) representations into atom sequences using RDkit and Graph Convolutional Networks (GCN). The Transformer decoder learns the interactive features between proteins and compounds, drawing from the protein sequence representations generated by the encoder and the atom sequence representations derived from SMILES. The final interaction feature vector is then passed to subsequent fully connected layers. Ultimately, the model returns the probability of interactions between compounds and proteins.

**2.3 CapBM-DTI**

CapBM-DTI [[10](#_ENREF_10)] is an innovative capsule network-based framework designed to address the challenges associated with accurately predicting drug-target interactions (DTIs) in the context of drug design and discovery. Unlike many existing computational methods for DTIs, CapBM-DTI overcomes limitations such as the lack of experimentally verified negative datasets, inaccurate molecular feature representation, and ineffective DTI classifiers. It achieves this by introducing two experimentally validated datasets and employing advanced techniques for feature extraction. CapBM-DTI leverages pre-trained bidirectional encoder representations from transformers (BERT) for contextual sequence feature extraction from target proteins, aided by transfer learning. Simultaneously, it utilizes the message-passing neural network (MPNN) for extracting 2-D graph features from compounds, resulting in robust and accurate DTI predictions. This model demonstrates its efficacy across diverse DTI datasets, including those from human (*Homo sapiens*) and worm (*Caenorhabditis elegans*) species, as well as subsets comprising new compounds, new proteins, and new pairs. Notably, CapBM-DTI showcases superior generalization capabilities and has potential applications in virtual screening, as evidenced by a case study on treating COVID-19. Its distinctive features include the establishment of experimentally validated negative datasets, the use of ProtBert for protein sequence vectorization through transfer learning, and the incorporation of capsules to capture hierarchical relationships, collectively outperforming existing state-of-the-art DTI prediction tools on multiple datasets.

**3 Signature-based drug repositioning algorithms**

In DrugRepoBank, six methods (GSEAweight0 [[11](#_ENREF_11)], GSEAweight1[[11](#_ENREF_11)], GSEAweight2 [[11](#_ENREF_11)], KS (Kolmogorov–Smirnov) statistic [[12](#_ENREF_12)], XSum (eXtreme Sum) score [[13](#_ENREF_13)] and ZhangScore [[14](#_ENREF_14)]) are utilized to measure the association between drug signature and disease signature by RCSM R package (https://github.com/Jasonlinchina/RCSM) [[15](#_ENREF_15)].

**3.1 GSEAweight method**

Gene Set Enrichment Analysis (GSEA), a Kolmogorov-Smirnov-like method, contains three calculation schemes for weighted KS enrichment statistic (ES): GSEAweight0, GSEAweight1, and GSEAweight2, with power (p) of gene expression of fold change (FC) is equal to 0, 1, and 2, respectively. Enrichment score (ES) reflects the degree of enrichment of gene set (S) in a ranked gene list (L={g_1_, g_2_, ..., g_N_}). The calculation of ES starts from the first gene along the gene list L. If a gene of L can be found in S, add a statistic; and if it cannot be found in S, reduce a statistic.

The calculation of enrichment score (ES):

1. $P_{hit}(S,i) = \sum_{j=1}^{i} \frac{\left| {FC}_{g_{j}} \right|^{p}}{\sum_{j=1}^{N} \left| {FC}_{g_{j}} \right|^{p}} , g_{j}\in S$; i=1, 2, ..., N; FC=fold change of gene expression; p=0,1 or 2.
2. $P_{miss}(S,i) = \sum_{j=1}^{i} \frac{1}{(N-Ns)} ,\left( g_{j}\in L \right)\cap\left( g_{j}\notin S \right)$; i=1, 2, ..., N; N=the number of genes in L; N_S_=the number of genes in S.
3. ES = the maximum deviation from 0 of P_hit_-P_miss_.

If ES(p)_up_ and ES(p)_down_ have different algebraic signs, the similarity score of the drug-disease pair:

$$GSEAweight(p)={ES(p)}_{\mathrm{up}}-{ES(p)}_{\mathrm{down}}$$

ES(p)_up_=the ES(p) score between Disease_up_ and Drug_complete_. ES(p)_down_=the ES(p) score between Disease_down_ and Drug_complete_. Disease_up_=top N up-regulated genes from the replicate-consensus signature of disease. Disease_down_=top N down-regulated genes from the replicate-consensus signature of disease. Drug_complete_=complete replicate-consensus signature of drug.

Otherwise,

$GSEAweight(p)=$0

**3.2 KS (Kolmogorov–Smirnov) statistic**

KS (Kolmogorov–Smirnov) statistic is similar to the GSEAweight method. The calculation of KS starts from a ranked gene list (L= {G_1_, G_2_, ..., G_N_}) and a gene set (S={g_1_, g_2_, ..., g_m_}). Then, build a vector V of each gene’s position based on gene list L. Besides, sort the genes in S by ascending order, where j=1, 2, ..., m.

$$a=\max_{j=1}^{m}\left[ \frac{j}{m}-\frac{V(j)}{N} \right]$$

$$b=\max_{j=1}^{m}\left[ \frac{V(j)}{N}-\frac{(j-1)}{m} \right]$$

$$KS=\left\{ \begin{aligned} a, if a>b, \\ -b, if b>a. \end{aligned} \right.$$

If KS_up_ and KS_down_ have different signs, the similarity score of the drug-disease pair:

$$\mathrm{KS}_{S}=\mathrm{KS}_{\mathrm{up}}-\mathrm{KS}_{\mathrm{down}}$$

KS_up_=the KS score between Disease_up_ and Drug_complete_; KS_down_=the KS score between Disease_down_ and Drug_complete_. Disease_up_=top N up-regulated genes from the replicate-consensus signature of disease. Disease_down_=top N down-regulated genes from the replicate-consensus signature of disease. Drug_complete_=complete replicate-consensus signature of drug.

Otherwise,

$$\mathrm{KS}_{S}=0$$

**3.3 XSum score**

XSum (The eXtreme Sum) score is focused on the top genes ranked by fold changes of gene expression.

For drug-disease pair:

XSum_up_=Disease_up_ ⋂Drug_changed_

XSum_down_=Disease_down_ ⋂Drug_changed_

$$XSum=sum(\mathrm{XSum}_{\mathrm{up}})-sum(\mathrm{XSum}_{\mathrm{down}})$$

Disease_up_=top N up-regulated genes from the replicate-consensus signature of disease. Disease_down_=top N down-regulated genes from the replicate-consensus signature of disease. Drug_changed_=top N up-regulated and top N down-regulated genes in drug.

**3.4 ZhangScore**

ZhangScore is designed to address scenarios involving disordered gene signatures, where rank-based weights are evenly distributed among all genes within the signature. For similarity score of drug-disease pair by ZhangScore method:

$$C(R,q)=\sum_{i=1}^{m} \mathrm{Drug}_{\mathrm{complete}}(g_{i})q(g_{i})$$

$$ZhangScore=\frac{C(\mathrm{Drug}_{\mathrm{complete}},q)}{\sum_{i=1}^{m} (n-i+1)}$$

Drug_complete_=complete replicate-consensus signature of drug. q refers to a compared gene signature, where q=(Disease_up_) ⋃ (Disease_down_). Disease_up_=top N up-regulated genes from the replicate-consensus signature of disease. Disease_down_=top N down-regulated genes from the replicate-consensus signature of disease. g_i_=the i^th^ gene in Drug_complete_. q(g_i_)=1 if gene g_i_ is up-regulated, it is equal to -1 if g_i_ is down-regulated; Drug_complete_(g_i_) is this gene’s signed rank in Drug_complete_. m is the length of gene signature q. n represents the length of Drug_complete_.

**4 Network-based drug repositioning algorithm**

DRviaSPCN (version 0.1.4), an R software package, is employed for predicting potential drug repositioning based on a subpathway (SP) crosstalk network [[16](#_ENREF_16)]. In this process, DrugRepoBank utilizes cancer signatures and employs the *CalCentralityScore* function to compute the centrality scores of SPs, which can reflect crosstalk effects. The resulting output provides information on SubPathID, SubPathway, Size, Gene, Centralscore, Pvalue, and FDR. Subsequently, the *Optimaldrugs* function is used to calculate the drug enrichment score (DES), and a threshold is set to filter out the most promising therapeutic drugs. DrugRepoBank opts for selecting the top 10 SPs from the ranked SP list (topcut=10), employs a significance threshold of 0.01 for screening SPs (pcut=0.01), performs 1000 permutations for calculations (nperm=1000), and utilizes unweighted methods to calculate the score. Moreover, the *Disease2SPheatmap* function is executed to visualize a heatmap of the disease-regulated subpathways with a p-value less than 0.05 (pcut=0.05).

DrugSim2DR (version 0.1.1) is a computational R package designed for drug repositioning in the context of specific diseases by utilizing a network-based approach. This methodology integrates drug-related information, RNA-seq data from diseased conditions and Gene Ontology (GO) functional annotations. To use DrugSim2DR, we starts with employing the *CalDEscore* function, which calculates a DEscore. This function requires input of a gene expression matrix where the samples are labeled (In oue study, “1” representing cancer samples and “0” denoting normal samples in each cancer types). The DEscore quantifies differential expression patterns between cancer and normal states. Subsequently, the *DrugReposition* function is key to identifying candidate drugs for repositioning. We feed into this function the DEscore results obtained from *CalDEscore*. Additionally, this function takes parameters such as nperm, indicating the number of permutations to assess significance, and r, which stands for the restart probability in the random-walk algorithm employed. By default, we set nperm to 1000 and r to 0.9.

**5 Web interface detail**

As shown in Figure S8, we provide DrugRepoBank with a user-friendly web interface for data presentation, search, and visualization. There are two main modules in DrugRepoBank: the “Prediction” module and the “Literature” module. The “Prediction” module aims to achieve the goal of drug repositioning by identifying new relationships between drugs and diseases or drugs and targets through multiple algorithms based on three search engines (Drug Search (Figure S8A), Target Search (Figure S8B) and Disease Search (Figure S8C)).

Drug Search not only provides basic information about drugs and known associations with targets and diseases but also offers information on new targets and diseases for the drug through drug-drug similarity-based methods (Figure 3A left; Figure S7A left), target-target similarity-based method (Figure 3A right; Figure S7A middle), and AI-based methods (Figure 3B; Figure S7A right). Drug Search includes four parts: (a) Drug search box. Search predicted drug repositioning candidates by Drug name, DrugBank ID, or PubChem compound ID of chemical compounds. (b) Basic information about the drug, such as drug type, structure, SMILE, therapeutic class, drug target (link to Target search for target-target similarity prediction), and so on. (c) Similarity-based drug repositioning: (i) The drug-drug similarity table presents drugs that exhibit high similarity to the queried drug, as predicted by six similarity-based methods. (ii) The drug-target-disease-side effect network aids users in visualizing new potential targets and potential therapeutic indications for the queried drug. (d) AI-based drug repositioning: (i) The drug-target interaction prediction table displays potential interactions between the queried target and drugs, as predicted by 3 AI-based methods. (ii) The drug-target-disease network aids users in visualizing new potential targets for the queried drug and their corresponding potential therapeutic indications.

Target Search not only offers basic information about targets and known associations with drugs and diseases but also provides predictions of new drugs binding with the target to treat new diseases through drug-drug similarity-based methods (Figure 3A left; Figure S7B left), target-target similarity-based method (Figure 3A right; Figure S7B middle), and AI-based methods (Figure 3B; Figure S7B right). Target Search includes four parts: (a) Target search box. Search target proteins by Uniprot ID or Gene Symbol. (b) Basic information about the target, including target type, sequence, function, binding drugs (link to Drug Search for drug-drug similarity prediction), and so on. (c) Similarity-based drug repositioning: (i) The target-target similarity table displays the similarity between the queried target and other targets. (ii) The drug-target-disease network assists users in visualizing new potential drugs for the queried target to potentially treat new diseases. (d) AI-based repositioning: (i) Target-drug interaction prediction Table is predicted by three artificial intelligence-based methods. (ii) The drug-target-disease network visualizes the predicted drugs that can interact with the queried target to potentially treat new diseases.

Disease Search provides differential gene expression and functional analysis information for disease signatures and can also discover disease-drug associations through signature-based (Figure 3C; Figure S7C up) and network-based methods (Figure 3D; Figure S7C down) to facilitate drug repositioning. Disease Search includes three parts: (a) Disease search box. Select the disease (cancer) type directly. (b) Disease (cancer) signatures: (i) Disease signature information including disease (cancer) name, number of up-and down-regulated genes, as well as the number of cancer and normal samples. (ii) Differentially expressed genes analysis includes the generation of a volcano plot and a heatmap. (iii) Pathway analysis includes KEGG and GO analysis of up-regulated genes, down-regulated genes, and combined DEGs. (iv) The whole expression gene table contains information on all genes, such as fold change, p-value, adjusted p-value, and other relevant statistics. (c) The Signature-based drug repositioning: (i) The Signature-based drug repositioning table is generated using six signature-based methods for prediction. (ii) The details of each entry for signature-based drug repositioning, including drug, cell line, dose, time, detailed score, and so on. (d) Network-based Drug Repositioning: (i) Network-based drug repositioning table is created by employing two network-based predictive approaches. (ii) The subpathway analysis table displays the subpathways regulated by cancer in detail, such as subpathway name, subpathway size, subpathway genes, central score, p-value, and FDR. (iii) Subway analysis heatmap helps users visually observe the subpathways regulated by the disease.

The “Literature” module is an experimentally validated drug repositioning part through a manual curation approach from the PubMed database, intending to provide literature support for existing repositioning drugs and uncover patterns in the discovery of repurposed drugs. Literature Search includes two parts: (a) Literature table includes the repositioned drug name, old target, old indication, new indirect target, new indication, PMID, etc. (b) Literature evidence contains *in silico*, *in vitro*, *in vivo*, and clinical evidence, supporting sentences, article information, and more.

**Reference**

1. Cao Y, Charisi A, Cheng L-C et al. ChemmineR: a compound mining framework for R, Bioinformatics 2008;24:1733-1734.

2. Pagès H, Aboyoun P, Gentleman R et al. Biostrings: Efficient manipulation of biological strings, R package version 2019;2:10.18129.

3. Yu G, Li F, Qin Y et al. GOSemSim: an R package for measuring semantic similarity among GO terms and gene products, Bioinformatics 2010;26:976-978.

4. Sancho L. BioCor: Functional similarities. R package version, 2019.

5. Wang JZ, Du Z, Payattakool R et al. A new method to measure the semantic similarity of GO terms, Bioinformatics 2007;23:1274-1281.

6. Tsubaki M, Tomii K, Sese J. Compound–protein interaction prediction with end-to-end learning of neural networks for graphs and sequences, Bioinformatics 2019;35:309-318.

7. Chen L, Tan X, Wang D et al. TransformerCPI: improving compound–protein interaction prediction by sequence-based deep learning with self-attention mechanism and label reversal experiments, Bioinformatics 2020;36:4406-4414.

8. Vaswani A, Shazeer N, Parmar N et al. Attention is all you need, Advances in neural information processing systems 2017;30.

9. Mikolov T, Chen K, Corrado G et al. Efficient estimation of word representations in vector space, arXiv preprint arXiv:1301.3781 2013.

10. Huang Y, Huang H-Y, Chen Y et al. A Robust Drug–Target Interaction Prediction Framework with Capsule Network and Transfer Learning, Int J Mol Sci 2023;24:14061.

11. Subramanian A, Tamayo P, Mootha VK et al. Gene set enrichment analysis: a knowledge-based approach for interpreting genome-wide expression profiles, Proceedings of the National Academy of Sciences 2005;102:15545-15550.

12. Lamb J, Crawford ED, Peck D et al. The Connectivity Map: using gene-expression signatures to connect small molecules, genes, and disease, Science 2006;313:1929-1935.

13. Cheng J, Yang L, Kumar V et al. Systematic evaluation of connectivity map for disease indications, Genome medicine 2014;6:1-8.

14. Zhang S-D, Gant TW. A simple and robust method for connecting small-molecule drugs using gene-expression signatures, BMC bioinformatics 2008;9:1-10.

15. Pallotto C, Sbrana F, Ripoli A et al. Daptomycin-based aminoglycoside-sparing therapy for streptococcal endocarditis: a retrospective multicenter study, J Chemother 2020:1-5.

16. Wu J, Li X, Wang Q et al. DRviaSPCN: a software package for drug repurposing in cancer via a subpathway crosstalk network, Bioinformatics 2022;38:4975-4977.
